# Disruption of lysosomal proteolysis in astrocytes facilitates midbrain organoid proteostasis failure in an early-onset Parkinson’s disease model

**DOI:** 10.1038/s41467-024-44732-2

**Published:** 2024-01-10

**Authors:** Gustavo Morrone Parfitt, Elena Coccia, Camille Goldman, Kristen Whitney, Ricardo Reyes, Lily Sarrafha, Ki Hong Nam, Soha Sohail, Drew R. Jones, John F. Crary, Alban Ordureau, Joel Blanchard, Tim Ahfeldt

**Affiliations:** 1 Nash Family Department of Neuroscience at Mount Sinai, New York, NY USA; 2 Ronald M. Loeb Center for Alzheimer’s Disease at Mount Sinai, New York, NY USA; 3 Friedman Brain Institute at Mount Sinai, New York, NY USA; 4 Black Family Stem Cell Institute at Mount Sinai, New York, NY USA; 5 Aligning Science Across Parkinson’s (ASAP) Collaborative Research Network, Chevy Chase, MD USA; 6 Department of Artificial Intelligence & Human Health, Icahn School of Medicine at Mount Sinai, New York, NY USA; 7 Department of Pathology, Molecular, and Cell-Based Medicine at Mount Sinai, New York, NY USA; 8 Cell Biology Program, Sloan Kettering Institute, Memorial Sloan Kettering Cancer Center, New York, NY USA; 9 Metabolomics Core Resource Laboratory, NYU Langone Health, New York, NY USA; 10 Present Address: Department of Neuroscience, Genentech, Inc., South San Francisco, CA 94080 USA; 11 Present Address: Recursion Pharmaceuticals, Salt Lake City, UT USA

**Keywords:** Astrocyte, Parkinson's disease, Neural ageing

## Abstract

Accumulation of advanced glycation end products (AGEs) on biopolymers accompanies cellular aging and drives poorly understood disease processes. Here, we studied how AGEs contribute to development of early onset Parkinson’s Disease (PD) caused by loss-of-function of DJ1, a protein deglycase. In induced pluripotent stem cell (iPSC)-derived midbrain organoid models deficient for DJ1 activity, we find that lysosomal proteolysis is impaired, causing AGEs to accumulate, α-synuclein (α-syn) phosphorylation to increase, and proteins to aggregate. We demonstrated these processes are at least partly driven by astrocytes, as DJ1 loss reduces their capacity to provide metabolic support and triggers acquisition of a pro-inflammatory phenotype. Consistently, in co-cultures, we find that DJ1-expressing astrocytes are able to reverse the proteolysis deficits of DJ1 knockout midbrain neurons. In conclusion, astrocytes’ capacity to clear toxic damaged proteins is critical to preserve neuronal function and their dysfunction contributes to the neurodegeneration observed in a DJ1 loss-of-function PD model.

## Abstract (web summary)

The protein DJ1, encoded by the PARK7 gene, is causally linked to development of early-onset PD. Here the authors observed that the loss of DJ1 function in midbrain organoids led to astrocyte dysfunction, impairing protein clearance, accumulation of α-synuclein.

## Introduction

Aging is the strongest risk factor for developing neurodegenerative diseases such as Alzheimer’s Disease (AD) and Parkinson’s Disease (PD)^1^. Accordingly, investigating how biological mechanisms of aging are interconnected with the progression of neurodegenerative diseases is an active research area with significant therapeutic potential. Recently, glycation, a process in which aldehyde metabolites and nucleophiles become attached to biopolymers through non-enzymatic reactions, has come into focus as a disease-driving mechanism. The accumulation of such advanced glycation end products (AGEs) on nucleotides, lipids, and proteins is known to damage cell function and is a feature of normal aging^2^. However, aberrant AGE accumulation has also been linked to multiple human pathologies, including aging-related neurodegenerative diseases such as PD. Both genetic and environmental factors contribute to the development of PD^3^, presenting challenges for identifying disease mechanisms that can be targeted therapeutically^4^. Recently, however, mutations in the α-synuclein gene (*SNCA*), the first identified causal mutations in PD^3^, were reported to impair proteome maintenance and cause protein aggregation^5^, highlighting protein quality control and autophagy as convergent disease-driving pathways in sporadic PD.

The protein DJ1, encoded by the *PARK7* gene, is causally linked to the development of early-onset PD by loss-of-function (LOF) mutations^6,7^, but how the reduced function of DJ1 contributes to PD pathogenesis is not understood. Because a conserved cysteine residue in DJ1 is known to be frequently oxidized, it was initially suggested that DJ1 can sense the oxidative state of the cell^8,9^. Indeed, highly oxidized sulfonated (−SO_3_^−^) forms of DJ1 are associated with its inactivation and are increased in the cortex of PD patients when compared to age-matched controls^8,10^. More recently, several studies have established that DJ1 has glyoxalase activity and may function as a deglycating enzyme that protects DNA, proteins, or lipids from harmful glycation, although some controversy remains about DJ1 substrates and activity levels^11–16^. In addition, studies using human-induced pluripotent stem cell (iPSC) models demonstrate that DJ1 impacts on oxidative stress pathways in neurons, particularly dopamine oxidation and autophagy pathways^17,18^. Collectively, these findings suggest that decreased DJ1 activity resulting from (−SO_3_^−^) oxidation may contribute to PD pathogenesis.

Cell death in PD is restricted to discrete neuronal populations in a few brain areas, and more specifically to dopaminergic neurons (DNs) in the *substantia nigra* (SN), which has led to a neuro-centric view of PD pathology^19^. However, in recent years, genome-wide association studies have increasingly implicated glial-associated genes in PD^20^. These findings are consistent with post-mortem analysis of PD cases, which often identifies astrocytes with a mild increase of GFAP in the SN, and accumulation of α-syn and PACRG in intracellular inclusions, unique features compared to the abundant astrogliosis observed in other neurodegenerative diseases^21–23^. More recently, a scRNA-seq analysis identified a population of CD44/S100A6-high reactive astrocytes in the midbrain of PD patients^24^. In addition, dysregulation of glial neurometabolic coupling and neuro-immune interactions were reported to have a crucial role in PD initiation and the death of the most vulnerable dopaminergic neurons^25–29^. Together, these observations suggest that astrocytes have a causal role in PD pathogenesis.

Although DJ1 is abundantly expressed in the CNS, it’s most highly expressed in astrocytes of the cortex and SN^8^. In animal models, DJ1 activity in astrocytes is protective against chemical-induced lesions to DNs of the SN^30,31^. Conversely, DJ1 knockout (KO) in mouse astrocytes leads to an exacerbated inflammatory response and impaired lesion repair^32^. Thus, although DJ1 has a well-established role as a protective protein in response to increased oxidative stress levels, more recently, its glyoxalase activity and role as a deglygating enzyme has been described. These findings propose that glycation stress is the core mechanism that underlies disease initiation and progression in DJ1 loss of function (LOF) early-onset PD.

The development of human midbrain brain organoids (hMIDOs) enabled the recapitulation of human midbrain tissue features such as mature TH-positive neurons and neuromelanin accumulation^17,33^. hMIDOs recapitulate PD-related phenotypes in various PD mutations in long-term cultures such as reduction in TH^+^ dopaminergic and accumulation of α-syn oligomers and phosphorylated forms^17,34^. Together with the scalability and stability of organoid models, hMIDOs make an ideal model for long-term studies and drug screening for PD pathology.

Here, we leverage a comprehensive panel of assays to investigate metabolic function in patient-derived human brain tissue and DJ1 LOF organoid iPSC models. We show that protein quality control pathways in astrocytes are defective in PD-associated DJ1 LOF. Accordingly, when DJ1 is missing or mutated, AGE accumulation and a-syn aggregation ultimately cause DN death. Our findings unravel the pathogenic role of astrocytes in aging and PD to uncover potential therapeutic strategies.

## Results

### DJ1 KO human midbrain organoids have PD-associated α-syn phenotypes

To study how DJ1 LOF impacts cellular processes in the absence of potential contributions from PD-associated genetics, we generated homozygous and heterozygous DJ1 knockout iPSC lines via CRISPR-mediated genome editing of BJSIPS iPSC line (originally derived from a healthy non-PD male). We confirmed the DJ1 KO via Sanger sequencing and Western blotting (Fig. S1A–B). In addition, we obtained a healthy male donor-derived hiPSC line KOLF 2.1J^35^ and an isogenic line harboring the PD-associated DJ1 L166P LOF point mutation. We selected two clones which we hereafter refer to as L166P-1 and L166P-2.

**Fig. 1:**
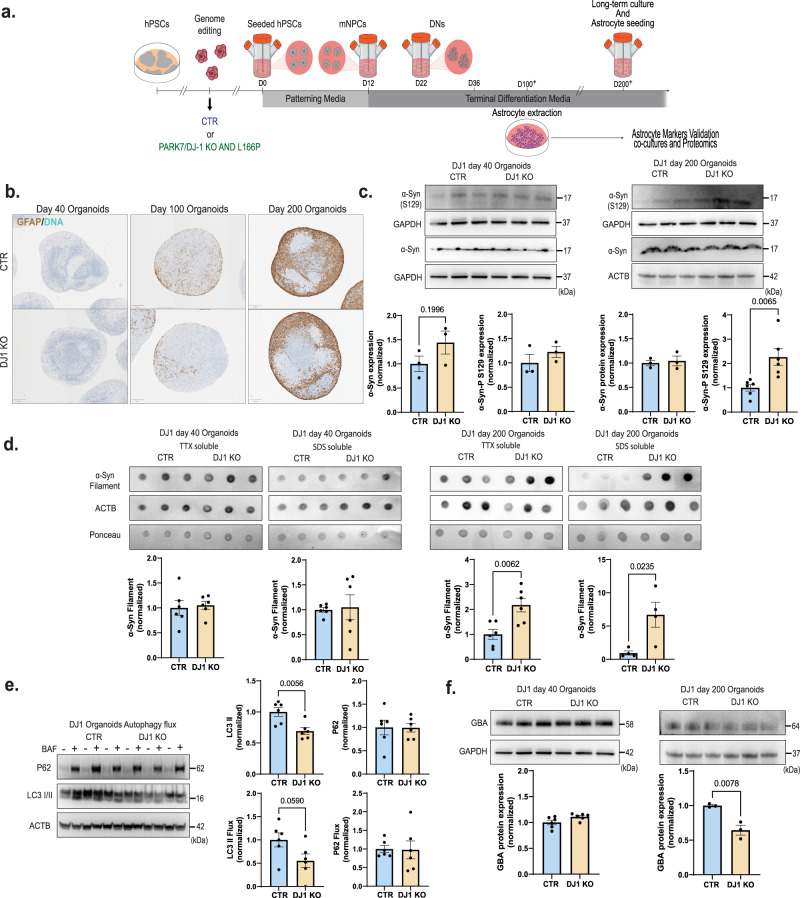
DJ1 KO human midbrain organoids α-syn and autophagy phenotypes. **a** Micrography GFAP staining of CTR and DJ1 KO day 40, 100, and 200 midbrain organoids. **b** Midbrain differentiation and astrocytes extraction protocol schematic. **c** Immunoblots for α-syn, phospho-α-syn (S129) (*n* = 3, Two-tailed *t*-test), and corresponding loading controls actin (ACTB) and GAPDH for CTR and DJ1 KO day 40 (α-syn *n* = 3, phospho-α-syn (S129), *n* = 3) and day 200 midbrain organoids (α-syn *n* = 3; phospho-α-syn (S129), *n* = 6). **d** Dot blots for oligomeric α-syn and actin (ACTB)/Ponceau loading control for CTR and DJ1 KO day 40 TTX soluble (*n* = 6); SDS soluble fractions (*n* = 6) and day 200 TTX soluble (*n* = 6) and SDS soluble fractions (*n* = 3) in midbrain organoids. **e** Immunoblots for LC3 I/II, P62, actin (ACTB) loading control in BAF – and + treated CTR and DJ1 KO day 100 midbrain organoids, graphs report LC3 I/II basal (*n* = 6), LC3 I/II flux (*n* = 6), P62 basal (*n* = 6), P62 flux (*n* = 6). **f** Immunoblots for GBA and ACTB/GAPDH loading control for CTR and DJ1 KO day 40 (*n* = 6) and day 200 (*n* = 3) midbrain organoids. All data are represented in mean ± S.E.M, data points are individual well differentiation, and the *p*-value was reported on the graph highlighted comparison. For all the comparisons, a Two-tailed *t*-test was applied. Panels **c** and **f** share the same loading controls. All measurements were taken from distinct samples.

To model the effects of DJ1 LOF on midbrain PD pathology, we generated human midbrain organoids (hMIDOs) using an established midbrain patterning protocol^17,36^ (Fig. 1a). Consistent with previous work, hMIDOs expressed midbrain markers FOXA2, LMX1A, and the mature midbrain marker NURR1, and generated TH^+^ neurons. At day 40, we found no differences in the expression of FOXA2, LMX1A, and NURR1 in DJ1 KO hMIDOs compared to control organoids (Fig. S1C). In contrast, we detected fewer NURR1-positive cells in organoids generated from the two DJ1 L166P clones (L166P-1 and L166P-2) (Fig. S1C). By day 100, astrocytes emerged, as reflected by GFAP expression, which became strongly increased by day 200 (Fig. 1b). In addition, the hMIDOs present extensive arborization when stained with a MAP2 marker at day 100 compared to day 40, indicating neuronal maturation. We confirmed the presence of mature dopaminergic neurons in organoids by day 100 by staining with GIRK2 and by measuring dopamine levels by mass spectrometry at day 200. We observed a significant reduction in the number of GIRK2 positive cells in the DJ1 KO (Fig. S1E), although dopamine levels did not differ between control and KO organoids (Fig. S1D).

The aggregation of the α-synuclein protein in the midbrain is a hallmark of PD^37^. Consistent with this phenotype, we found that aged DJ1 KO hMIDOs contained significantly increased levels of monomeric phosphorylated α-syn at day 200 relative to isogenic control midbrain organoids (Fig. 1c). In contrast, we observed no significant difference in α-syn phosphorylation at day 40, suggesting that day 40 and 200 midbrain organoids represent early and late disease stages (Fig. 1c). Given that phosphorylation of α-synuclein at the S129 residue is correlated with α-syn turnover and accumulation in PD patients’ brains^38^, we next assessed α-synuclein aggregation via western blotting. In day 40 organoids, we did not observe significant differences in α-synuclein between DJ1 KO and controls (Fig. 1d). However, in TTX- and SDS-soluble fractions of day 200 DJ1 KO midbrain organoids, levels of aggregated α-syn had increased (Fig. 1d).

Lysosome pathways are essential to multiple forms of autophagy and are known to process α-syn monomers and aggregates. We consistently detect lysosomal function as a dysregulated pathway using proteomics analysis of human embryonic stem cells (hESCs) and day 35 hESC-derived hMIDOs lacking DJ1^17^. Here, we re-analyzed our proteomics dataset using pathway analysis to identify a wider range of cellular processes potentially related to lysosomal dysfunction (Fig. S1F). To investigate whether accumulation of α-synuclein aggregates in DJ1 KO hMIDOs was due to alterations in autophagy, we treated day 100 hMIDOs with bafilomycin A1 (BAF), which blocks lysosomal activity by inhibiting lysosome acidification. We then quantified levels of the autophagosome marker LC3 II, which is generated through lipidation of LC3 I and essential for autophagosome formation. In BAF-treated samples, our analysis shows that LC3 II flux levels were not reduced significantly in DJ1 KO relative to CTR hMIDOs although a significant reduction in the basal levels was observed (Fig. 1e). Similarly, accumulation of the autophagy substrate P62 was unchanged in BAF-treated KO hMIDOs (Fig. 1e). In additional, we observed depletion of the mature form of the lysosomal enzyme GBA on day 200 hMIDOs but no alterations at day 40 (Fig. 1f), consistent with earlier reports in stem-cell-derived neuronal PD models^39,40^. These results point to a failure of the autophagy system to compensate for the higher generation of aggregates in DJ1 KO hMIDOs, overall causing the accumulation of oligomeric α-syn.

### DJ1 KO midbrain organoids accumulate protein glycation damage

**Fig. 2:**
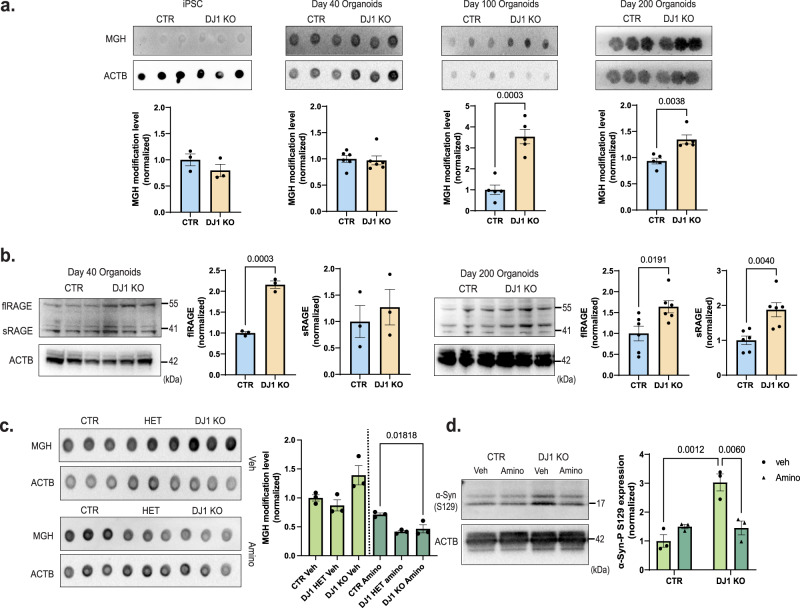
DJ1 KO midbrain organoids have increased protein glycation. **a** Dot blots for MGH protein modification and actin (ACTB)/Ponceau loading control for CTR and DJ1 KO iPSCs (*n* = 3), day 40 (*n* = 6), 100 (*n* = 5), and 200 (*n* = 5) midbrain organoids (Two-tailed *t*-test was used for mean comparisons). **b** Immunoblots for flRAGE, for CTR and DJ1 KO day 40 (*n* = 3) and day 200 (*n* = 6) and sRAGE CTR and DJ1 KO day 40 (*n* = 3) and day 200 (*n* = 6) in midbrain organoids; actin (ACTB) was used as loading control (Two-tailed *t*-test was used for mean comparisons). **c** Dot blots for MGH protein modification and actin (ACTB) loading control in vehicle (Veh) or aminoguanidine (Amino) treated CTR and DJ1 KO day 100 midbrain organoids (*n* = 3, Two-way ANOVA followed by Tukey’s for the multiple comparisons test). **d** Immunoblots for phospho-α-syn (S129) and actin (ACTB) loading control in vehicle (Veh) or aminoguanidine (Amino) treated CTR and DJ1 KO day 200 midbrain organoids (*n* = 3, Two-way ANOVA followed by Bonferroni’s for the multiple comparisons test). All data are represented in mean ± S.E.M, data points are individual well differentiation, and the *p*-value was reported on the graph highlighted comparison. All measurements were taken from distinct samples.

Oxidative damage, which compromises both proteome stability and cell viability^41,42^, has been proposed to drive neurodegeneration through accumulation of advanced glycation end-products (AGEs)^43,44^. In PD patients, AGEs and their corresponding RAGE receptors accumulate in the SN and cortex^45^, potentially due to DJ1 dysfunction, given its reported role as a deglycase that maintains AGE homeostasis^15^. Here, we quantified Methylglyoxal-derived hydroimidazolone (MGH) levels, an initial advanced glycation modification, in iPSCs and human midbrain organoids via dot blots. Although MGH levels were equivalent in DJ1 KO and control in both iPSCs and day 40 midbrain organoids, MGH protein glycation accumulated significantly in day 100 and day 200 DJ1 KO hMIDOs relative to controls (Fig. 2a).

RAGE expression is a sensitive biomarker for the presence of AGEs^46^, therefore we were intrigued to observe a significant increased full-length and soluble RAGE protein at day 200 DJ1 KO, and partially in day 40 midbrain organoids (Fig. 2b). The increased levels of RAGE in day 40 organoids could indicate that early glycation damage is below levels of detection by MGH blotting or that non-MGH glycation damage is present. When we used the Seahorse XF assay, we found no change in the glycolysis rate (ECAR) or glycolytic capacity between the hMIDO DJ1 KO and control midbrain organoids at day 40 or 100 (Fig. S2A). Similarly, mass spectrometry analysis did not reveal any differentially expressed metabolites correlated with increased reactive glycation or glycolysis (Fig. S2B). Collectively, these data suggest that the increase in AGEs observed in DJ1KO midbrain organoids is likely a direct effect of the lack of DJ1’s activity. However, mechanisms by which DJ1 caused the accumulation of AGEs could not be concluded from this set of experiments.

Next, we asked whether AGE accumulation in the midbrain contributes to classical PD phenotypes such as α-synuclein aggregation and lysosomal dysfunction. When we treated day 100 midbrain organoids for forty days with aminoguanidine (Amino), a scavenger of reactive carbonyl groups, we detected a significant reduction of MGH glycated proteins in DJ1 KO midbrain organoids relative to amino-treated DJ1 CTR organoids (Fig. 2c). In parallel with reduced MGH, we also observed significantly decreased α-synuclein phosphorylation (S129) in Amino-treated DJ1 KO organoids (Fig. 2d). In contrast, there was no significant difference in α-synuclein phosphorylation in Amino-treated control organoids (Fig. 2d). The lack of alterations in DJ1 HETs was expected due to the autosomal recessive nature of the PD *PARK7* mutation. Because toxic reactive dicarbonyls that attack guanine residues in DNA can cause double-strand breaks^15^, we measured phospho-H2A.X (P-H2A.X) levels as an indirect readout for DNA damage response. We observed a consistent increase in the P-H2A.X levels in DJ1 KO iPSCs and hMIDO groups with no alterations in DJ1 HET iPSCs compared to CTR. However, no alteration in basal H2A.X levels was found among all the groups (Fig. S2C). Overall, these experiments suggest that AGEs may influence abundance and phosphorylation of α-synuclein.

### Astrocyte DJ1 LOF causes non-cell-autonomous neuronal toxicity and impairs astrocytic metabolic support of midbrain neurons

Astrocytes are the principal glycolytic cell type in the brain and are, therefore, at a higher risk of acquiring glycation damage than neurons and other brain cell types. As astrocytes are essential for neuronal homeostasis and degradation of neuronal-derived damaged lipids and proteins such as α-syn^28,47^, we next investigated astrocyte function in midbrain organoids. To this end, we developed a protocol for isolating astrocytes from mature midbrain organoids (day 100+) and maintaining them in 2D culture. Isolated astrocytes were immunoreactive for canonical astrocyte markers CD44, EAAT2, S100B, and GFAP (with no significant difference among genotypes) (Fig. S3A) and functionally responsive to ATP stimulation measured using the calcium sensor GCaMP7 (Fig. S3B). In addition, organoid-derived astrocytes were double positive for the midbrain makers NURR1 and FOXA2 and co-stained with CD49f and CD44 expression, confirming their midbrain identity^48^ (Fig. S3C).

**Fig. 3:**
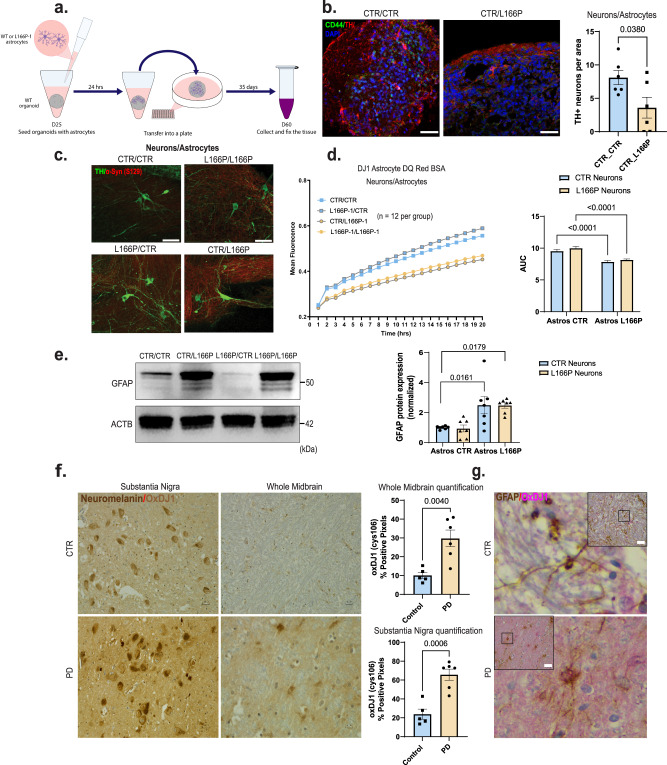
Astrocyte DJ1 LOF are reactive and produce toxicity in the midbrain. **a** Astrocytes seeding procedure schematic. **b** Images of organoids seeded with astrocytes stained for TH (Red), CD44 (green), and DAPI and quantification of TH+ positive neurons per organoid area (*n* = 6, Two-tailed *t*-test was used for mean comparisons). **c** 40× images of day 50 NPC/Astrocyte co-cultures stained for TH (green), phospho-α-syn (S129) (Red). **d** DQ BSA proteolysis live imaging in mixed genetic neuron/astrocytes co-cultures (*n* = 12, Two-way ANOVA followed by Tukey’s for the multiple comparisons test). **e** Western blot for GFAP and actin (ACTB) loading control for Neurons/Astrocytes co-cultures and quantification (*n* = 7, Two-way ANOVA followed by Tukey’s for the multiple comparisons test). **f** 40× micrography of the *substantia nigra* and midbrain of PD patients and age-matched controls showing OxDJ1 staining in light brown. QuPAth of OxDJ1 positive pixel quantification (CTR *n* = 5 and PD *n* = 6, Two-tailed *t* test was used for mean comparisons). **g** 40× micrography GFAP positive midbrain astrocytes (brown) and OxDJ1 (red). All data are represented in mean ± S.E.M. Scale bars, 50 µm for (**c**), (**f**), and (**g**); 70 µm for (**b**). Data points are individual well differentiation or individual patients, and the *p*-value was reported on the graph highlighted comparison. All measurements were taken from distinct samples.

To investigate non-cell-autonomous effects of DJ1 loss of function mediated by astrocytes, we seeded day 25 hMIDO with mature L166P-1 or control astrocytes (Fig. 3a). We collected and fixed the tissue at day 60 when endogenous astrocytes were still not present in the organoids. We confirmed the graft efficiency with staining for the astrocyte marker CD44 (Fig. 3b). We found that adding L166P-1 astrocytes to WT midbrain organoids significantly reduced the number of TH-positive neurons (Fig. 3b). In addition, we co-cultured astrocytes with midbrain NPCs that, by day 50, expressed TH and featured mature neuronal morphology (Fig. 3a). Phospho-α-syn (S129) was present in DJ1 LOF astrocytes groups (L166P-1/L166P-1 and CTR/L166P-1) (Fig. 3c). When we analyzed total proteolysis capacity using DQ™ Red BSA, and found that the proteolysis capacity of the DJ1 LOF NPCs was significantly increased when co-cultured with CTR astrocytes (L166P-1/CTR) relative to L166P-1/L166P-1 groups (Fig. 3d). In addition, co-cultures of DJ1 LOF astrocytes with CTR NPCs (CTR/ L166P-1) proteolysis levels were disrupted when compared to the CTR/CTR group (Fig. 3d), suggesting that proteolytic clearance may be impaired in DJ1 LOF astrocytes. We also analyzed the protein levels of GFAP in astrocytes co-cultured with midbrain NPCs. When L166P-1 astrocytes were cultured with CTR or L166P-1 NPCs, a significant increase in GFAP levels was observed, demonstrating the inflammatory potential of DJ1 LOF in astrocytes (Fig. 3e).

To investigate these relationships in the human brain, we quantified cell-type expression of DJ1 in the midbrain of a cohort of PD patients (STable 1). We observed that GFAP-positive astrocytes were positive for ox-cys106 DJ1, which correlates with increased DJ1 activity. (Fig. 3g). We also measured DJ1 activation levels by quantifying ox-cys106 DJ1 in the substantia nigra (SN) and midbrain. When compared to age-matched controls, PD patients had significantly increased levels of ox-cys106 DJ1 in the SN and whole midbrain (Fig. 3f, STable 1). Collectively, this analysis suggests that DJ1 has a prominent neuroprotective role in astrocytes and that PD-associated DJ1 LOF variants contribute to neurodegeneration via astrocytes.

### DJ1 LOF astrocytes have a pro-inflammatory phenotype and accumulate aggregated proteins

**Fig. 4:**
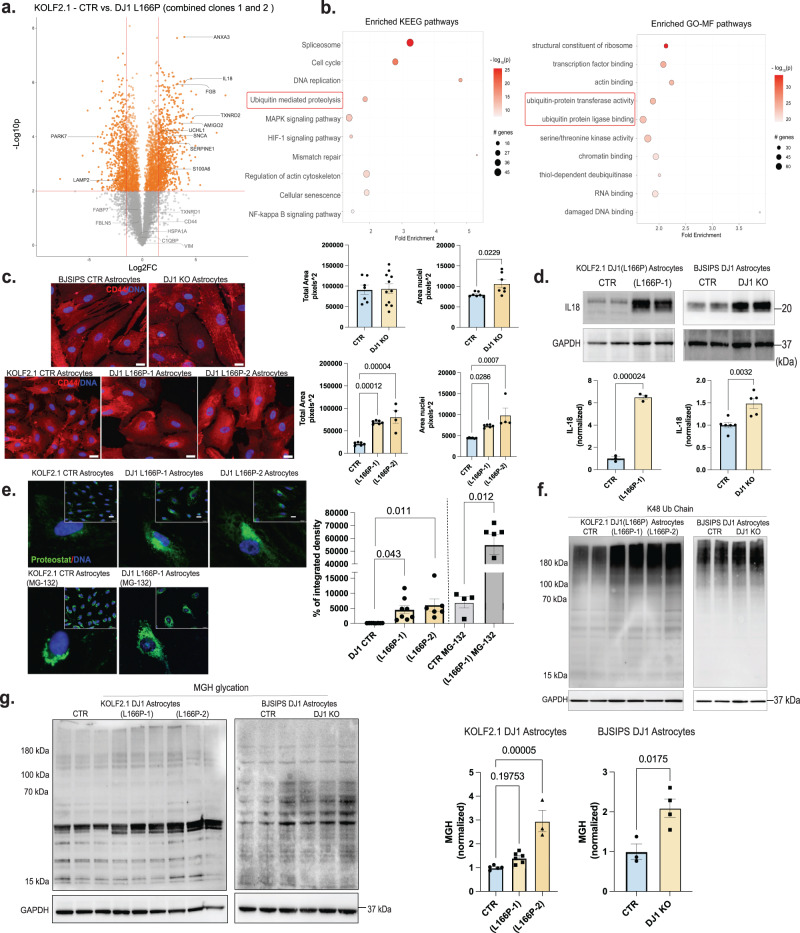
Pro-inflammatory and aggregated proteins are increased in DJ1 LOF astrocytes. **a** Volcano plot representation of TMT-labelled proteomics in KOLF 2.1 J (*n* = 3) and DJ1 L166P-1 (*n* = 3) and L166P-2 (*n* = 3) midbrain astrocytes with selected proteins labeled (fold prioritization of LogFC vs. control of 1.5; Welch’s *t*-test was used for the comparisons). **b** KEEG and FO-MF pathways enrichment analysis using pathfindR (*p* values were adjusted by the Bonferroni method) showing the selected top 10 terms. **c** 40× micrography of CD44 (red) and DAPI (blue) staining in BJ-SiPS CTR (*n* = 7) and KO (*n* = 11) astrocytes (Two-tailed *t*-test was used for mean comparisons) and KOLF 2.1 J CTR (*n* = 6), DJ1 L166P-1 (*n* = 6), and L166P-2 (*n* = 4) astrocytes (One-way ANOVA followed by Tukey’s for the multiple comparisons test). Quantification of cell total area based on CD44 staining and nuclear area based on DAPI staining. **d** IL18 in BJ-SIPS CTR (*n* = 6) and KO (*n* = 5) astrocytes and KOLF 2.1 J CTR (*n* = 3), DJ1 L166P-1 (*n* = 3) astrocytes (Two-tailed *t*-test was used for mean comparisons). **e** 40x micrography and quantification proteostat fluorescence levels in KOLF 2.1 J CTR (*n* = 8), L166P-1 (*n* = 8) and L166P-2 (*n* = 6) (One-way ANOVA followed by Tukey’s for the multiple comparisons test). 40× micrography and quantification proteostat fluorescence levels in KOLF 2.1 J CTR + MG132 (*n* = 4), DJ1 L166P (*n* = 60) (Two-tailed *t*-test was used for mean comparisons). **f** Immunoblots for K48 ubiquitin chain and **g** MGH protein modification in KOLF 2.1 J CTR (*n* = 5), L166P-1 (*n* = 6), and L166P-2 (*n* = 3) (One-way ANOVA followed by Tukey’s for the multiple comparisons test). MGH protein modification in BJ-SIPS CTR (*n* = 3) and KO (*n* = 4) (Two-tailed *t*-test was used for mean comparisons). All data are represented in mean ± S.E.M., data points are individual well differentiation, and the *p*-value was reported on the graph highlighted comparison. Proteomics data related to KN073-96 dataset. Scale bars, 20 µm. All measurements were taken from distinct samples.

To identify pathways that contribute to non-cell-autonomous phenotypes mediated by DJ1 LOF astrocytes, we performed global TMT-proteomics and phospho-proteomics in astrocytes derived from 2 clones (L166P-1 and L166P-2) of KOLF 2.1J DJ1 L166P and their respective CTRs. The MS proteomic analysis identified ~8000 different proteins expressed in all samples. PCA analysis showed that the 2 DJ1 L166P clones clustered together and separated from the CTR. Therefore, we combined the two DJ1 L166P clones for the final analyses (Fig. S4A–B). In total, we identified 1192 differentially expressed proteins in the combined DJ1 L166P dataset (Fig. 4a, *P*-value of <0.01, logFC > 1.5 or <−1.5) indicating a significant proteome alteration that included upregulation of inflammatory/reactivity-related proteins such as ANXA3, FGB, AMIGO2, and SERPINE1 and pro-inflammatory interleukins IL32 and IL18 as the most highly differentially upregulated proteins in the DJ1 L166P astrocytes (Fig. 4a). When we probed our dataset for PD risk proteins, we found that α-synuclein and UCHL1 were increased, together with the mitochondrial redox balancing enzyme TXNRD2 (Fig. 4a).

To identify molecular pathways altered in DJ1 L166P astrocytes, we performed an enrichment analysis using the Pathfinder package, which considers protein-protein interactions. Among the top terms, we found the ubiquitin/proteasome system (UPS), cytoskeleton modification, and inflammatory responses (Fig. 4b, SData1). We then performed phospho-proteomics to identify differentially expressed phosphorylation sites and predict the most active kinases in DJ1 L166P astrocytes (Fig. S4D). We identified more than 10,000 different phosphorylated residues with 4709 differentially expressed based on this analysis. Using PhosR kinase activity prediction analysis, we identified the CDK isoforms and MAPKP8 as the most likely phosphorylating proteins involved in regulating the cytoskeleton and inflammation, including the innate immune response regulator IRAK4 and other related proteins. In addition, when we plotted the CDK family with respect to differentially regulated phosphorylation sites, we observed auto-phosphorylation of CDK1 and increased phosphorylation in cytoskeletal proteins such as MAP4, MAP1B, MAPT, and septin9 (Fig. S4D). We also conducted pathway enrichment analysis for the CDK isoforms and MAPK8 using the top phosphorylation sites from your phosphoproteomics dataset. This analysis demonstrated their involvement in cytoskeleton remodeling (Fig. S4D).

Given that reactive astrocytes undergo key morphological change known as hypertrophy^49^, we next performed immunostaining for the glial cell body marker CD44 and Hoechst 33342 to evaluate nuclear morphology (Fig. 4c). In the full DJ1 KO, we did not detect any alterations in the cell body area among the groups. However, the nuclear area was increased in DJ1 KO astrocytes, and both nuclear size and cell body area were increased in DJ1 L166P astrocytes (Fig. 4c). We also detected increased IL18 expression in both DJ1 KO and DJ1 L166P astrocytes, which is consistent with our proteomics analysis (Fig. 4d).

Our proteomics analysis of DJ1 L166P astrocytes identified alterations in proteolysis and an increase in α-synuclein. To relate these phenotypes with PD pathology, we performed multiple functional assays. In the presence of the ProteoStat dye (which binds and fluoresces in the presence of protein aggregates), fluorescence intensity was significantly increased in DJ1 L166P astrocytes, indicating an accumulation of protein aggregates (Fig. 4e). After treatment with the proteasome inhibitor MG-132, aggregation increased in both DJ1 L166P lines, and CTR astrocytes reached levels comparable to those found in UT L166P (Fig. 4e). DJ1 KO and DJ1 L166P astrocytes also expressed higher levels of K48 ubiquitin chain proteins at high molecular weight, which is characteristic of protein aggregates (Fig. 4f). These observations indicate a higher UPS activity in DJ1 L166P combined with an inability to degrade protein aggregates. When we integrated the proteomics data with the aggregation risk scores (generated ZaggSC and TANGO^50^) for each differential protein, we identified several proteins enriched in DJ1 L166P cells that were at increased risk for neurodegeneration-associated aggregation, such as APOA1, JADE1, and SERP1 (Fig. S4C). In addition, similar to DJ1 KO hMIDOs, DJ1 LOF in astrocytes also led to a significant increase in levels of MGH-glycated, with the exemption to clone L166P-1 (Fig. 4g). Altogether, these analyses suggest that proteome instability leads to inflammation, reactivity, and cytokine release in astrocytes.

### DJ1 loss of function impairs the lysosome, leading to accumulation of α-syn in astrocytes

**Fig. 5:**
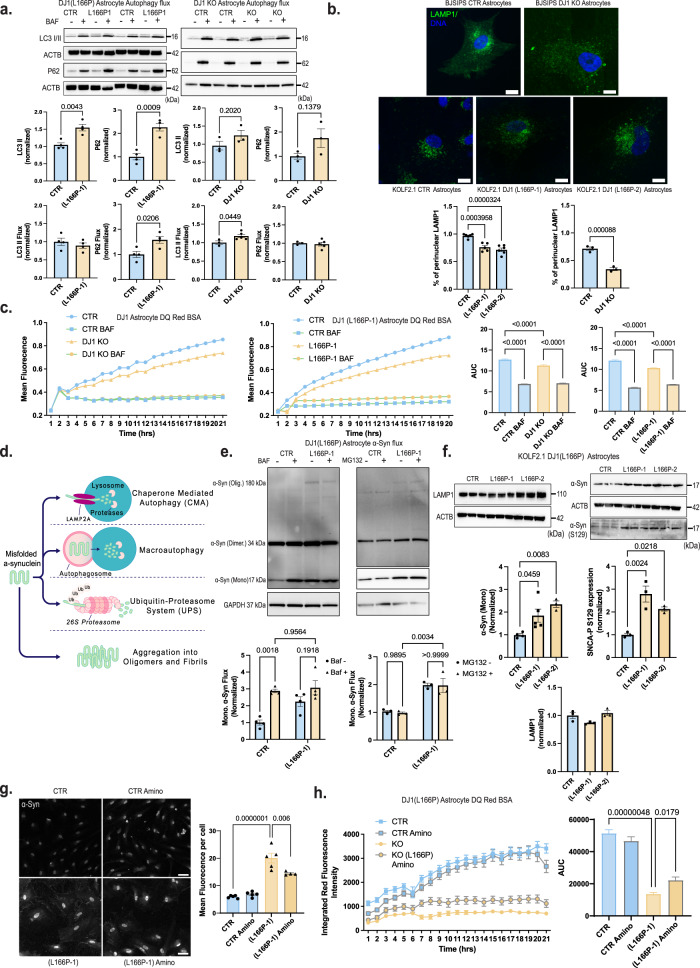
DJ1 loss of function impairs proteostasis in astrocytes. **a** Immunoblots for P62, LC3 I/II, actin (ACTB) loading control in BAF – and + treated CTR and DJ1 KO in KOLF 2.1 J and BJ-SIPS midbrain astrocytes. Immunoblots for LC3 I/II, P62, actin (ACTB) loading control in BAF – and + treated KOLF 2.1 J CTR, DJ1 L166P-1 astrocytes, graphs report LC3 I/II basal (*n* = 4), LC3 I/II flux (*n* = 4), P62 basal (*n* = 4), P62 flux (*n* = 4). Immunoblots for LC3 I/II, P62, actin (ACTB) loading control in BAF – and + treated CTR and DJ1 KO astrocytes, graphs report LC3 I/II basal (*n* = 3), LC3 I/II flux (CTR, *n* = 3 and DJ1 KO, *n* = 5), P62 basal (*n* = 3), P62 flux (CTR, *n* = 3 and DJ1 KO, *n* = 5) (Two-tailed *t*-test was used for mean comparisons). **b** LAMP1 (green) and DAPI (blue) staining of astrocytes 40x images. Quantification of LAMP1 distribution staining in BJ-SiPS CTR (*n* = 3) and KO (*n* = 3) astrocytes (Two-tailed *t*-test was used for mean comparisons) and KOLF 2.1 J CTR (*n* = 6), DJ1 L166P-1 (*n* = 5), and L166P-2 (*n* = 7) astrocytes (One-way ANOVA followed by Tukey’s). **c** DQBSA proteolysis live imaging assay treated CTR and DJ1 L166P-1 KOLF 2.1 J astrocytes (BAF −, *n* = 40, BAF + *n* = 7) or CTR and DJ1 KO BJ-SIPS (BAF −, *n* = 39, BAF + *n* = 8) treated with BAF- and BAF + astrocytes (Two-way ANOVA followed by Tukey’s). **d** Schematics showing α-syn degradation pathways. **e** Immunoblots for α-syn in BAF (*n* = 4) and MG132 (*n* = 3) – and + treated CTR and DJ1 L166P-1 KOLF 2.1 J astrocytes (Two-way ANOVA followed by Tukey’s). **f** Immunoblots for LAMP1 (*n* = 3), α-syn (CTR, *n* = 3; L166P-1, *n* = 5; and L166P-2, *n* = 3), phospho-α-syn (S129) (*n* = 3), and GAPDH loading control in and KOLF 2.1 J CTR, L166P-1, and L166P-2 astrocytes (One-way ANOVA followed by Tukey’s). **g** 40× images of total α-syn (gray) staining of astrocytes and quantification of vehicle (Veh) or aminoguanidine (Amino) treated astrocytes of CTR (CTR, *n* = 5; and CTR Amino, *n* = 5) or DJ1 L166P genotypes (L166P-1, *n* = 5; and L166P-1 Amino, *n* = 4) (Two-way ANOVA followed by Tukey’s). **h** DQBSA proteolysis live imaging assay of vehicle (Veh) or aminoguanidine (Amino) treated astrocytes of CTR or DJ1 L166P-1 genotypes (*n* = 24, Two-way ANOVA followed by Tukey’s). Scale bars, for b-15 µm; g-40 µm. All data are represented in mean ± S.E.M., data points are individual well differentiation, and the *p*-value was reported on the graph highlighted comparison. All measurements were taken from distinct samples.

Degradation of aggregated proteins, defined as autophagic flux (the rate of autophagic degradation) is upregulated in early stages of neurodegenerative diseases^42,51^. We therefore sought to determine whether the accumulation of protein aggregates observed in DJ1 LOF astrocytes resulted from impaired autophagy or reduced ability to increase autophagy flux. When we analyzed autophagy flux in both DJ1 LOF astrocyte lines, we found that the levels of lipidated LC3 II increased significantly in L166P-1 BAF- treated group relative to untreated controls with no alteration in the DJ1 KO astrocytes (Fig. 5a). However, when we compared BAF- groups with their respective BAF+ treated controls, we observed a 10% significant increase in the DJ1 KO group in LC3 II flux levels, and no alteration in the L166P-1 group (Fig. 5a) suggesting that the formation of the autophagosome is not impaired in both DJ1 LOF lines. Next, we analyzed P62 flux to evaluate the ability of the autophagosome to degrade cargo. We found that levels of P62 increased significantly in the L166P-1 baseline and flux groups, and no alteration was found for DJ1 KO groups relative to untreated groups was observed (Fig. 5a).

Next, we assessed perinuclear localization of the lysosome, an essential feature for chaperone-mediated autophagy (CMA) activity and protein degradation^52^. In both DJ1 KO and DJ1 L166P astrocytes, we observed a decrease in perinuclear endo/lysosomes, as detected by LAMP1 and DAPI immunostaining, relative to the CTR group (Fig. 5b). Consistently, when we quantified proteolytic function using live imaging of DQ™ Red BSA, which releases fluorescence upon proteolysis, we detected impaired proteolysis in both DJ1 KO and L166P astrocytes (Fig. 5c). Inhibition of the lysosome with BAF decreased fluorescence intensity, demonstrating that BSA proteolysis is largely performed by the lysosome (Fig. 5c). To deepen our understanding of the impaired lysosomal function, we next evaluated the presence of the early and late lysosomal damage markers GAL3 and K48 and the repair marker CHM4b^53^. In the DJ1 L166P KO groups, percentages of LAMP1-positive puncta that were also positive for GAL3 and K48 were both increased, and we also observed an increase in K48 intensity per area relative to control (Fig. S5A). The percentage of LAMP1-positive puncta for the repair marker CHMP4b also increased in the DJ1 L166P KO groups (Fig. S5A). Collectively, these data reveal that although the endo/lysosomal system in DJ1 L166P KO cells sustains more damage, it is still capable of triggering repair systems.

Toxic soluble and insoluble forms of α-syn are believed to accumulate in midbrain cells when degradation of aggregated forms through macro-autophagy fails to meet the demand of the elevated α-syn flux. α-Syn degradation is primarily performed by CMA and with the UPS as the preferred mechanism for aggregated forms (Fig. 5d). We analyzed the autophagy capacity to degrade monomeric α-syn, and found that monomeric α-syn in BAF-treated CTR increased, whereas the DJ1 L166P KO group had a decreased flux (Fig. 5e). Untreated DJ1 KO astrocytes also had significantly higher synuclein monomers than control astrocytes (Fig. 5e). In contrast, the amount of synuclein monomers in BAF-treated control astrocytes did not decrease, suggesting that DJ1 KO impairs the lysosomal clearance of synuclein monomers (Fig. 5e). Although the UPS is known to contribute to α-syn degradation^54^, levels of α-syn did not significantly increase when control or DJ1 KO astrocytes were treated with the proteasomal inhibitor MG132, suggesting that proteasomal activity has minimal contribution to a-syn degradation in astrocytes (Fig. 5e). In addition, the monomeric unmodified and S129 phosphorylated forms of α-syn increased in the DJ1 L166P lines compared to the CTR, while no alteration was observed in the endo/lysosome marker LAMP1 (Fig. 5f).

**Fig. 6:**
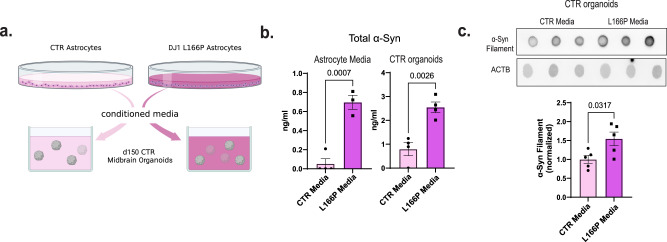
Astrocyte DJ1 loss of function conditioned media leads to α-syn increase in midbrain organoids. **a** Experimental design for conditioned media experiment, created with BioRender.com. **b** Competitive ELISA test for α-syn in media (CTR Media, *n* = 3; and L166P-1 Media, *n* = 4) and CTR organoid tissue (*n* = 4). **c** Dot blots for oligomeric α-syn and actin (ACTB) loading control for CTR (*n* = 5) and L166P (*n* = 5) astrocyte media treated midbrain organoids. All data are represented in mean ± S.E.M., data points are individual well differentiation, and the *p*-value was reported on the graph highlighted comparison. All measurements were taken from distinct samples. For all the comparisons, a Two-tailed *t*-test was applied.

Based on our data thus far, we hypothesized that AGE damage is at least partly responsible for the reduced lysosomal proteolysis observed in DJ1 LOF astrocytes. When we treated astrocytes with the Methylglyoxal (MGO) scavenger Amino for 10 days, we noticed that lysosomal proteolysis was significantly improved in L166P KO astrocytes (Fig. 5h). Consistently, total α-syn levels were significantly reduced in treated relative to untreated KO L166P cells with no differences observed in treated controls (Fig. 5g). In addition, we tested if the treatment could reverse the DJ1 LOF DNA damage measured indirectly by phospho-H2AX. The levels of phospho-H2AX were significantly increased in L166P-1 astrocytes relative to CTR, with no differences between the L166P-1 vehicle versus L166P-1 Amino-treated astrocytes (Fig. S3F). This data indicates that Amino specifically relieves glycation damage in KO L166P cells. To evaluate glycation stress more specifically, we treated DJ1 KO astrocytes with the reactive dicarbonyl MGO, the main cause of glycation in live cells, and measured apoptotic nuclei and poly-caspase activation. Relative to untreated control cells, we detected significantly higher numbers of apoptotic nuclei and higher levels of poly-caspase in untreated DJ1 KO astrocytes (Fig. S3D). In addition, MGO treatment significantly increased the number of apoptotic nuclei in DJ1 KO, while no change in cell death hallmarks was observed in CTR astrocytes (Fig. S3D). When we examined proteolysis, we found decreased levels in DJ1 L166P astrocytes and that these levels were even further reduced by MGO treatment (Fig. S3E). To identify whether soluble factors released by the DJ1 LOF astrocytes could be toxic to neurons, we performed a conditioned media experiment on CTR organoids (Fig. 6a). We detected a significant increase in α-syn in both DJ1 L166P astrocytes media and media-treated CTR hMIDOs (Fig. 6b). We also detected a significant increase in soluble α-syn oligomers in hMIDOs treated with L166P astrocyte media, which indicates release of toxic soluble factors by L166P astrocytes (Fig. 6c). Altogether, our data show that DJ1 loss of function reduces lysosomal capacity, resulting in the accumulation of toxic forms of synuclein and increased neuronal cell death.

## Discussion

Failed protein quality control and proteome damage are common features of several neurodegenerative diseases^17,18,55^. Here, we show that DJ1 activity is essential to prevent accumulation of toxic damaged proteins in the midbrain. Although we did not detect alterations in soluble α-syn monomers as a consequence of DJ1 LOF, we detected an increase in soluble oligomers, indicative of dynamic relationships between α-syn oligomers and oligomerized or aggregated proteins that may be routed via different degradation processes. At a cellular level, our data show that DJ1 LOF or mutations hinder lysosomal processing of α-syn in astrocytes. Consequently, soluble α-syn oligomers aggregate into toxic forms, resulting in direct oxidative damage to the proteome of midbrain astrocytes and subsequent activation of astrocyte-derived inflammation signals that further aggravate pathology. These data deepen and extend from the established view of astrocytes in PD inflammation.

Glycation is a form of oxidative protein damage commonly observed in diabetes, a risk factor for developing PD^56,57^. We observed accumulation of MGH, an early form of glycation damage derived from MGO, in non-aggregated proteins in both hMIDOs and isolated astrocytes. Nonetheless, non-aggregated glycated forms are thought to be degraded by the proteasome, and their build-up can trigger ER stress^58,59^. In what way DJ1 protects from protein glycation damage is still unclear. Early work proposed that DJ1 integrates with the glycation stress system through enzymatic function^12^, participates in MGO degradation, or prevents permanent glycation damage by direct repair of early glycation products^14,15^. In patient-derived iPSC neurons bearing DJ1 mutation glycation-related products were found elevated^60^. However, DJ1 deficiency failed to enhance neuronal cell death when challenged with MGO^60^. In the DJ1 KO organoids, we observe a progressive accumulation of glycated proteins starting at day 100. Glycolysis levels and MGO precursor metabolites were unaltered, which indicates a failure in MGO degradation or protein glycation repair. Conversely, presence of the carbonyl scavenger Amino decreased levels of MGH-modified proteins in both CTRs and DJ1 KO, validating its ability to prevent glycation stress, potentially by preventing the glycation stress-induced increase in α-syn phosphorylation. Altogether, these findings suggest that DJ1 participates in glycation metabolism, and DJ1 mutation contributes to PD by causing slow accumulation of deleterious glycation products.

In the absence of DJ1, levels of inflammatory cytokines IL18 and IL32 and downstream signal events such as IRAK4 phosphorylation were both increased. Consistently, the proteome analysis identified various upregulated proteins in DJ1 LOF astrocytes, such as ANXA3, S100A6, FGB, and SERPINE, while others, such as VIM and S100A10, were unchanged. These data reveal a unique astrocyte signature with relevance for PD-associated pathology, consistent with recent findings that reported glial cell activation upon scRNA-seq analysis of the human midbrain in PD patients^24^. In a broad number of cells including astrocytes, cytoskeleton modification and changes in morphological features to sense, mobilize, or invade are the main characteristics of inflammation. Our proteome and phosphorylation pattern indicates an elevated modification of microtubule-associated proteins, which can explain the observed altered morphology of the DJ1 LOF astrocytes and the increase in nuclear size and cell body hypertrophy we observed in cells with the L166P mutation. We found differential expression of MAP4, MAP1B, MAPT, and other proteins related to cytoskeleton modification. In addition, our prediction analysis identified the CDK kinase family, reported to phosphorylate cytoskeleton-associated proteins and other related proteins^61^, as the top active kinases predicted to phosphorylate the enriched residues.

Many PD genes participate in convergent pathways that are altered in the pathology^62^. Therefore, the finding that additional familial PD genes, such as *SNCA* and *UCHL1*, were upregulated in LOF DJ1 astrocytes was in line with these observations. We reported these changes in isolated astrocytes extracted from organoids, which points to the crucial contribution of astrocytes to specific PD phenotypes, such as α-syn accumulation. However, we cannot exclude a previously reported prion-like acquisition in which the spreading of α-syn throughout the hMIDOs causes damage^63^.

In addition to lysosomal changes, we also identified UPS system alterations in our enrichment pathway analysis, consistent with the increase in aggregated protein inclusions and the sensitivity of the DJ1 mutation cell lines to proteasome inhibition. These relationships indicate the presence of ongoing protein damage that continuously needs to be repaired by the proteasome and lysosome systems. Although we only detected a mild alteration of autophagy in the L166P lines, α-syn oligomers were formed due to impaired lysosomal degradation of α-syn. Our observations indicate a varied phenotype depending on the type of mutation studied, with the DJ1 L166P mutation harbouring the most deleterious phenotypes when compared to the DJ1 KO lines. The DJ1 L166P mutation generates an inactive truncated DJ1 protein which misfolds and therefore constantly needs to be degraded by the UPS system, which could further exacerbate the disease-associated phenotypes^64,65^. In agreement, we found an increase in ATF6 in the DJ1 L166P cells, which increases activation due to the accumulation of misfolded products, leading to the induction of inflammatory gene networks^66^.

Dysregulated networks common to early-onset and sporadic PD converge on proteostasis failure, causing accumulation of aggregated proteins^17,18^. Based on our studies in the early-onset PD DJ1 model, we hypothesize that accumulation of glycated products, which overburden and damage protein degradation and repair systems, ultimately leads to proteostasis failure. In addition, due to their high non-aerobic glycolysis, astrocytes would be particularly vulnerable to this oxidative damage. In conclusion, we provide evidence that astrocytes dysfunction, glycation and widespread protein aggregation are fundamental phenotypes in familial DJ1-linked PD, pointing to strategies for developing more effective therapeutics.

## Methods

### Generation of isogenic knockout lines

Human iPSCs were cultured in standard conditions, and before nucleofection cells were pre-treated for 1 h. with 10 μM Rhok inhibitor. 4 × 10^6^ cells were dissociated using Accutase. Cells were pelleted and resuspended in 800 μl PBS^–/–^ containing 5 μg px330 CRISPR DNA each and transferred into nucleofection cuvettes. Nucleofection was performed using either the P3 Nucleofector kit from Amaxa and the standard and program CB-150 or the primary P4 Nucleofector kit from Amaxa and the standard and program hiPSC CA-137. The iPSC lines used for the generation of the TdTomato DJ1 WT, HET and KO were from the BJSIPS background and the guides as described in Ahfeldt^17^. The iPSC lines used for the generation of the DJ1 L166P point mutation were from KOLF 2.1 J background^35^ (Pantazis et al., 2021). DJ1 L166P clones 1 and 2 were used in this study. The parental cell lines were karyotyped prior to the beginning of the experiments. Genotyping PCR was used to identify clones with homozygous or compound heterozygous deletions leading to truncations and frameshift mutations. Clones for all lines containing deletions were identified by Sanger sequencing. No ethical oversight was necessary for this study.

### Midbrain differentiation and organoid maintaining

hiPSCs were cultured in Stemflex^TM^ medium (ThermoFisher) at 37 °C, with 5% CO_2_ in a humidified incubator, as previously described^36^. For the organoid aggregation and differentiation, 125-ml disposable spinner flasks (Corning, VWR) were placed on a nine-position stir plate (Dura-Mag) at a speed of 65 rpm, as previously reported, starting with dissociated 40 × 10^6^ hPSCs in Stemflex + Rhok inhibitor Y- 27632 (4 μM). Differentiation was initiated when spheres reached 300–500 µm by dual-SMAD inhibition with SB431542 (R&D Systems, 10 μM), LDN193189 (Stemgent, 100 nM), B27-Vit A and N2 in DMEM-F12, to ensure the proper size range spheres were filtered using a set of 300 and 500 µm filters (pluriSelect). Midbrain-specific patterning for midbrain NPCs organoids was the addition of CHIR99021 (Stemgent, 3 μM), Purmorphamine (STEMCELL, 2 μM), and SAG (Abcam, 1 μM)^48^. Post patterning Neural maturation medium was DMEM F12 medium containing N2, B27-VitA, 20 ng/mL GDNF (R&D Systems), 20 ng/mL BDNF (R&D Systems), 0.2 mM ascorbic acid (Sigma), 0.1 mM dibutyryl cAMP (Biolong), 10 μM DAPT (Cayman Chemical). For long-term maintenance (after day 35), the spheres were transferred to ultralow attachment plates (Corning, VWR) at five spheres per ml of media. The medium for long-term culture was DMEM F12 medium containing N2, B27-VitA, 10 ng/ML GDNF (R&D Systems), 10 ng/mL BDNF (R&D Systems), 0.2 mM ascorbic acid (Sigma).

### Astrocyte differentiation and isolation

We derived astrocytic 2D cultures from large-scale 3D spin cultures, starting at day 90 using a protocol adapted from TCW et al.^67^. This involved dissociation and serial passaging of midbrain organoids under conditions that favor astrocyte growth. Cells are grown in 125 ml flasks containing hundreds of individual organoids totaling more than 4 × 10^8^ cells. Currently, the culture comprises various midbrain cell types including DNs, other neurons, progenitor cells, and astrocytic cell types. To isolate astrocytic progenitors, we gently triturated organoids in trypsin enzyme solution using a glass pipette to break up individual organoids into large chunks. After washing steps and pelleting of the organoids, we plated the suspension on 15 cm dishes coated with 0.1% gelatin. Cells are maintained in an astrocyte propagation medium (Astrocyte Medium, ScienCell #1801) for a week or until the first astrocytes are attached and start to divide. Cells were passaged to a maximum of P3. The maintaining and experimental media consisted of Advanced DMEM/F-12 (1 part, ThermoFisher 12634010), Neurobasal (1 part, ThermoFisher 21103049), B-27 Supplement, N-2 Supplement, MEM Non-Essential Amino Acids Solution (ThermoFisher 11140050), GlutaMAX (ThermoFisher 35050061), CNTF (10 ng/ml, PeproTech 450-13). For passaging, Astrocyte Medium was used on the first day and replaced by Astrocyte mature after 70% confluency was reached. For the conditioning media experiments, mature media was added to a confluent plate of astrocytes. After 3 days, the media was collected, filtered, and then added to day 150 hMIDOs.

### Midbrain organoid astrocyte seeding and co-culture

For the co-culture experiments, midbrain NPCs of day 14 after differentiation and day 100 astrocytes were plated in a 96-well plate in a 5:1 proportion. They were maintained in a post patterning neural maturation medium for 2 weeks and then transferred to a long-term culture medium for the experimental procedures. For the seeding procedure, 12 organoids were seeded with 50k astrocytes each in a V bottom plate (Corning), and serum was added to the media to support the astrocyte seeding for 72 h. After 48 h, organoids were transferred to a 96-well plate, one organoid per well, and maintained until day 60.

### Immunocytochemistry of fixed cells

Cells were fixed with 4% PFA for 20 min. Cells were blocked in 0.1% Triton X-100 (Sigma) in 5% horse serum/PBS, and then incubated in primary antibody (STable 2) (0.1% Triton X-100 in 5% horse serum/PBS) overnight at 4 °C. On the following day, cells were washed in PBS and incubated in species-specific fluorophore-conjugated Alexa fluor secondary antibodies and DAPI nuclear stain according to the manufacturer’s protocol. The imaging was performed using the High content imager CX7 (Thermo Fisher) with a HCS Studio 4.0 software, and phenotyping and quantifications were performed using ImageJ v1.53 or CellProfiler v3 softwares. For the statistical analysis, the average of at least three fields of the well was counted as a N value.

### Immunostaining and image analysis of sectioned spheres

Organoids were fixed with 4% PFA O/N and embedded in paraffin. Serial sections (4–6 μm) of paraffin-preserved midbrain organoid sections were prepared using a Leica RM2255 microtome, sections were placed on charged slides and baked overnight at 70 °C. IHC was performed on Ventana Benchmark XT. Antigen retrieval with CC1 (citric acid buffer) was performed for 1 h, followed by primary antibody incubation for 30 min (min.). A multimer secondary antibody was used for all samples. IHC sections were imaged using an Aperio VERSA 8 (Leica Biosystems, Wetzlar Germany) digital slide scanner and analyzed in QuPath (version 0.2.3, https://QuPath.github.io/).

### Immunohistochemistry of human tissue

Cases and controls brain samples were derived from the Mount Sinai Neuropathology Brain Bank. Inclusion criteria were individuals with a neuropathological diagnosis of Parkinson’s disease for cases and cognitively normal with no or only age-related neuropathological changes for controls. Formalin-fixed paraffin-embedded (FFPE) sections (5μm) were prepared from substantia nigra blocks, mounted on positively charged slides, and baked overnight at 70 °C. Immunohistochemistry (IHC) was performed on a Ventana Benchmark XT automatic staining platform (Roche Diagnostics, Indianapolis, IN) according to the manufacturer’s protocol with reagents and antibodies acquired from the same lot. Antigen retrieval was done using CC1 buffer (Tris/Borate/EDTA buffer, pH 8.0–8.5, Roche Diagnostics) for 1 h followed by primary antibody incubation. All primary antibodies were diluted in antibody dilution buffer (ABD24, Roche Diagnostics). Primary antibodies were incubated for 36 min (mOXDJ1, 1:400, Abcam) or 28 min (GFAP, 1:10, Ventana, 760-4345) followed by either 3,3’-diaminobenzidine (DAB) or alkaline phosphatase for visualization. For slides that were double-labeled, both DAB and alkaline phosphatase were used for visualization. All slides were counterstained with hematoxylin and coverslipped.

### Digital histopathologic analysis

For unbiased digital quantitative assessment, slides were imaged using an Aperio VERSA 8 (Leica Biosystems, Wetzlar Germany) digital slide scanner. Whole tissue sections and the substantia nigra were manually neuroanatomically segmented on whole slide images (WSI) and analyzed in QuPath (version 0.2.3, https://QuPath.github.io/). All analysis was batch-processed using a custom positive pixel-based analysis workflow that measured the percentage of positive pixels detected using a positive pixel classifier based on thresholded values for DAB intensity. All quantitative values were normalized to the area.

### Live-cell imaging to access lysosome function

Astrocyte monocultures or midbrain neuronal/astrocyte co-cultures were plated in the 96-well plates. Cells were incubated with DQ™ Red BSA, which emits red fluorescence upon proteolysis, for 30 min, washed 3 times with PBS, and kept in maintaining media throughout the assay. Bafilomycin A2 (100 nM, Sigma-Aldrich B1793) was added to specific wells to confirm the lysosomal nature of the proteolysis. Aminoguanidine (30 µM) was used for the reversal experiments. The plates were incubated for image acquisition in Incucyte® Live-Cell Analysis System for 21 h and images were analyzed in the Incucyte® software v2020C.

### Calcium Imaging in astrocytes

For GCaMP8s imaging, plated astrocytes in 2 cm gelatin-coated plates were placed on a Nikon Eclipse TE2000-U microscope, with a 10× objective. GCaMP8s was excited using a 480 nm (Mic-LED-480A, Prizmatix), an HQ480/40× excitation filter, a Q505LP dichroic mirror, and an HQ535/50 m emission filter (Semrock). Fluorescence was projected onto an sCMOS Zyla chamber camera (VSC-01910, Andor), and sampled at a rate of 4.7 fps with a frame exposure of 200 ms at 160 × 120 pixels (4 × 4 binning). The light source and sCMOS camera were controlled with the Nikon Elements software (NIS-Elements AR 5.20.01). The astrocytes were continuously perfused during fluorescence recording with ACSF with the following composition (in mM): NaCl 125, KCl 5, D-Glucose 10, HEPES-Na 10, CaCl_2_ 3.1, MgCl_2_ 1.3. The ATP treatment (100 μM) was controlled by a ValveBank8 II (AutoMate Scientific Inc.). ROI segmentation of GCaMP8s, raw fluorescence extraction, and background correction was performed with Nikon Elements software. ΔF/F was calculated using R-studio (R version 4.0.3).

### Western blotting and dot blot

Cell culture lysates were generated using RIPA buffer (Thermo Scientific), 1 or 2% SDS lysis buffer (10 mM tris, 150 mM NaCl, 1 mM EDTA) containing protease inhibitor cocktail (Thermo Scientific), and phosphatase inhibitor cocktail (Thermo Scientific). Protein concentration was estimated using the BCA assay (Pierce). For Western blot analysis, 20–40 µg total protein was denatured under reducing conditions in 4× Laemmli Sample Buffer (Bio-Rad) by heating for 10 min at 70 °C before loading onto a 10% Criterion TGX Precast gel (Bio-Rad), then transferred to a PVDF membrane (0.22 µm; Bio-Rad) using the iBlot 2 dry blotting system (Invitrogen). Membranes were blocked for 1 h at RT in 5% w/v non-fat milk (Santa Cruz) in TBS containing 0.1% v/v Tween-20 (Fisher Scientific; TBS-T). Membranes were then incubated in the indicated primary antibody (in 5% milk/TBS-T) overnight at 4 °C, washed 4 times in TBS-T, incubated in species-specific HRP-conjugated secondary antibody (in 5% milk/TBS-T) for 1 h at RT, and then washed 4 times in TBS-T. Membranes were subsequently developed with ECL Western blotting substrate (Pierce). Membranes were then washed once in TBS-T and stripped in stripping buffer (25 mM Glycine HCl, pH 2.0, and 1% w/v SDS) with vigorous shaking to remove primary and secondary antibodies, washed three times in TBS-T, and blocked for 1 h (in 5% milk/TBS-T) at RT before probing with the next primary antibody. Dot blots were performed in the same way as western blots but without the gel separation step. The primary antibodies are listed in STable 2.

### Proteomics—cell lysis and protein digestion

At the indicated times, cells were washed twice with ice-cold PBS and snap-frozen. Cell pellets were lysed with in-house RIPA buffer (50 mM HEPES, 150 mM NaCl, 1% sodium deoxycholate, 1% NP-40, 0.1% SDS, 2.5 mM MgCl_2_, 10 mM sodium glycerophosphate, 10 mM sodium biphosphate) containing in-house protease and phosphatase inhibitor cocktail), to produce whole-cell extracts. Whole-cell extracts were sonicated and clarified by centrifugation (16,000 × *g* for 10 min at 4 °C) and protein concentrations were determined by the Bradford assay. Protein extracts (40 µg) were subjected to disulfide bond reduction with 5 mM TCEP (room temperature, 10 min) and alkylation with 25 mM chloroacetamide (room temperature, 20 min). Methanol–chloroform precipitation was performed before protease digestion. In brief, four parts of neat methanol were added to each sample and vortexed, one part of chloroform was then added to the sample and vortexed, and finally three parts of water were added to the sample and vortexed. The sample was centrifuged at 8000 rpm for 5 min at room temperature and subsequently washed twice with 100% methanol. Samples were resuspended in 100 mM EPPS pH8.5 containing 0.1% RapiGest and digested at 37 °C for 16 h with trypsin at a 100:1 protein-to-protease ratio.

### Proteomics—Tandem Mass Tag labeling

Tandem Mass Tag (TMT and TMTpro) labeling of samples was carried out as followed. For total proteome analysis (40 µg of digested peptides), 8 μL of a 10 μg/μL stock of TMT reagent was added to samples, along with acetonitrile to achieve a final acetonitrile concentration of approximately 30% (v/v). Following incubation at RT for 1 h, the labeling efficiency of a small aliquot was tested for each set, and the reaction was then quenched with hydroxylamine to a final concentration of 0.5% (v/v) for 15 min. The TMT-labeled samples were pooled together at a 1:1 ratio. The total proteome sample was vacuum centrifuged to near dryness and subjected to C18 solid-phase extraction (SPE) (50 mg, Sep-Pak, Waters).

### Proteomics—off-line basic pH reversed-phase (BPRP) fractionation

Dried TMT-labeled sample was resuspended in 100 μl of 10 mM NH4HCO3 pH 8.0 and fractionated using basic pH reverse phase HPLC. Briefly, samples were offline fractionated over a 90 min run, into 96 fractions by high pH reverse-phase HPLC (Agilent LC1260) through an aeris peptide xb-c18 column (Phenomenex; 250 mm × 3.6 mm), with mobile phase A containing 5% acetonitrile and 10 mM NH4HCO3 in LC-MS grade H2O, and mobile phase B containing 90% acetonitrile and 10 mM NH4HCO3 in LC-MS grade H2O (both pH 8.0). The 96 resulting fractions were then pooled in a non-continuous manner into 24 fractions used for subsequent mass spectrometry analysis. Fractions were vacuum centrifuged to near dryness. Each consolidated fraction was desalted via Stage Tip, dried again via vacuum centrifugation, and reconstituted in 5% acetonitrile, and 1% formic acid for LC-MS/MS processing. For Phospho-peptides, dried peptides were fractionated according to the manufacturer’s instructions using High pH reversed-phase peptide fractionation kit (Thermo Fisher Scientific) for the final 6 fractions and subjected to C18 StageTip desalting prior to MS analysis.

### Proteomics—Fe2+-NTA phosphopeptide enrichment

Phosphopeptides were enriched using Pierce High-Select Fe2+-NTA phosphopeptide enrichment kit (Thermo Fisher Scientific, A32992) following the provided protocol. In brief, dried peptides were enriched for phosphopeptides and eluted into a tube containing 25 μL 10% formic acid (FA) to neutralize the pH of the elution buffer and dried down. The unbound peptides (flowthrough) and washes were combined, dried up, and subjected to basic pH reversed-phase fractionation (see method section) and used for the total proteome analysis (non-phosphorylated peptides).

### Proteomics—Total proteomics analysis

Mass spectrometry data were collected using an Orbitrap Eclipse Tribrid mass spectrometer (Thermo Fisher Scientific, San Jose, CA) coupled to an UltiMate 3000 RSLCnano system liquid chromatography (LC) pump (Thermo Fisher Scientific). Peptides were separated on a 100 μm inner diameter microcapillary column packed in-house with ~30 cm of HALO Peptide ES-C18 resin (2.7 µm, 160 Å, Advanced Materials Technology, Wilmington, DE) with a gradient consisting of 5–23% (0–75 min), 23–40% (75–110 min) (ACN, 0.1% FA) over a 120 min run at ~500 nL/min. For analysis, we loaded 3/10 of each fraction onto the column. Each analysis used TMT-MS2 based quantification, combined with the FAIMS Pro Interface (using previously optimized 3 CV parameters for TMT multiplexed samples^68^. The scan sequence began with an MS1 spectrum (Orbitrap analysis; resolution 120,000 at 200 Th; mass range 400−1500 *m/z*; automatic gain control (AGC) target 4 × 105; maximum injection time 50 ms). Precursors for MS2 analysis with desired charge state (z: 2–6) were selected using a cycle type of 1.25 s/CV method (FAIMS CV = −40/−60/−80). MS2 analysis consisted of high energy collision-induced dissociation (HCD) and was analyzed using the Orbitrap (resolution 50,000 at 200 Th; NCE 38; AGC 2 × 105; isolation window 0.5 Th; maximum injection time 172 ms). Monoisotopic peak assignment was used, precursor fit filter was used (80% fit) and previously interrogated precursors were excluded using a dynamic window (150 s ± 10 ppm). For TMTpro analysis, a similar setup was used with the following modifications. Each analysis used Multi-Notch MS3-based TMT quantification^69^, combined with a newly implemented Real-Time Search analysis software^70,71^. MS2 analysis consisted of collision-induced dissociation (quadrupole ion trap analysis; Rapid scan rate; AGC 1.0 × 104; isolation window 0.5 Th; normalized collision energy (NCE) 35; maximum injection time 35 ms). Monoisotopic peak assignment was used, precursor fit filter was used (70% for a fit window of 0.5 Th) and previously interrogated precursors were excluded using a dynamic window (180 s ± 10 ppm). Following the acquisition of each MS2 spectrum, a synchronous-precursor-selection (SPS) API-MS3 scan was collected on the top 10 most intense ions b or y-ions matched by the online search algorithm in the associated MS2 spectrum^70,71^. MS3 precursors were fragmented by high energy collision-induced dissociation (HCD) and analyzed using the Orbitrap (NCE 45; AGC 2.5 × 105; maximum injection time 200 ms, the resolution was 50,000 at 200 Th). The closeout was set at two peptides per protein per fraction so that MS3s were no longer collected for proteins having two peptide-spectrum matches (PSMs) that passed quality filters.

### Proteomics—Phospho-proteomics analysis

Mass spectrometry data were collected using an Orbitrap Eclipse Tribrid mass spectrometer (Thermo Fisher Scientific, San Jose, CA) coupled to an UltiMate 3000 RSLCnano system liquid chromatography (LC) pump (Thermo Fisher Scientific). Peptides were separated on a 100 μm inner diameter microcapillary column packed in-house with ~30 cm of HALO Peptide ES-C18 resin (2.7 µm, 160 Å, Advanced Materials Technology, Wilmington, DE) over a 155 min run at ~500 nL/min. For analysis, we loaded half of each fraction onto the column. Each analysis used the FAIMS Pro Interface (using previously optimized 3 CV parameters for TMT-labeled phosphopeptides) to reduce ion interference. The scan sequence began with an MS1 spectrum (Orbitrap analysis; resolution 120,000 at 200 Th; mass range 350−1500 *m/z*; automatic gain control (AGC) target 4 × 105; maximum injection time 50 ms). Precursors for MS2 analysis were selected using a cycle type of 1.25 s/CV method (FAIMS CV = −40/−60/−80). MS2 analysis consisted of high energy collision-induced dissociation (HCD) (Orbitrap analysis; resolution 50,000 at 200 Th; isolation window 0.5 Th; normalized collision energy (NCE) 38; AGC 2 × 105; maximum injection time 172 ms). Monoisotopic peak assignment was used, precursor fit filter was used (80% for a fit window of 0.5 Th) and previously interrogated precursors were excluded using a dynamic window (120 s ± 10 ppm)^72^.

### Proteomics—Data analysis

Mass spectra were processed using a Comet-based (2019.01 rev. 5) software pipeline^73^. Spectra were converted to mzXML and monoisotopic peaks were re-assigned using Monocle^74^. MS/MS spectra were matched with peptide sequences using the Comet algorithm along with a composite sequence database including the Human Reference Proteome (2020-01 - SwissProt entries only) UniProt database, as well as sequences of common contaminants. This database was concatenated with one composed of all protein sequences in the reversed order. Searches were performed using a 50 ppm precursor ion tolerance for analysis. TMT or TMTpro tags on lysine residues and peptide N termini (+229.162932 TMT; (+304.207 Da TMTpro) and carbamidomethylation of cysteine residues (+57.021 Da) were set as static modifications, while oxidation of methionine residues (+15.995 Da) was set as a variable modification. For the phosphorylation dataset search, phosphorylations (+79.966 Da) on Serine or Threonine were set as additional variable modifications. Peptide-spectrum matches (PSMs) were adjusted to a 1% false discovery rate (FDR). PSM filtering was performed using a linear discriminant analysis^75^, while considering the following parameters: XCorr (or Comet Log Expect), ΔCn (or Diff Seq. Delta Log Expect), missed cleavages, peptide length, charge state, and precursor mass accuracy. For protein-level comparisons, PSMs were identified, quantified, and collapsed to a 1% peptide false discovery rate (FDR) and then collapsed further to a final protein-level FDR of 1%. Moreover, protein assembly was guided by principles of parsimony to produce the smallest set of proteins necessary to account for all observed peptides. For TMT-based reporter ion quantitation, we extracted the summed signal-to-noise (S:N) ratio for each TMT channel and found the closest matching centroid to the expected mass of the TMT reporter ion (integration tolerance of 0.003 Da). Reporter ion intensities were adjusted to correct for the isotopic impurities of the different TMT reagents according to manufacturer specifications. Proteins were quantified by summing reporter ion signal-to-noise measurements across all matching PSMs, yielding a “summed signal-to-noise” measurement. For total proteome, PSMs with poor quality, MS3 spectra with 6 or more TMT reporter ion channels missing, or isolation specificity less than 0.8, or with TMT reporter summed signal-to-noise ratio that was less than 150 or had no MS3 spectra were excluded from quantification. Phosphorylation site localization was determined using the AScore algorithm. AScore is a probability-based approach for high-throughput protein phosphorylation site localization. Specifically, a threshold of 13 corresponded to 95% confidence in site localization.

Protein or peptide quantification values were exported for further analysis in Microsoft Excel, R package, and Perseus. Each reporter ion channel was summed across all quantified proteins and normalized assuming equal protein loading of all samples. Phospho-peptides were normalized to the corresponding protein abundance value (when available). Additional analysis was done using PathfindR v1.6.1, QIAGEN Ingenuity Pathway Analysis (IPA) v1.0, and PhosR v1.12R packages^76,77^. To predict the most active kinases, we obtained the significantly upregulated photo-peptides in the L166P dataset. We input the upregulated phopho-peptides list into kinase activity prediction tool to generate a score of activity based on based on Kim et al.^76^. A detailed step-by-step guide can be found in Kim et al.^78^. For the pathfindR analysis of the proteomics dataset, we included up and down-regulated proteins with a cut-off from the adjusted limma *p*-value of 0.05.

Supplemental data Tables list all quantified proteins as well as the associated TMT reporter ratio to control channels used for quantitative analysis.

### LC-MS/MS with the hybrid metabolomics and dopamine analysis

Organoids were subjected to an LCMS analysis to detect and quantify known peaks. A metabolite extraction was carried out on each sample based on a previously described method^79^. The LC column was a Millipore TMZIC-pHILIC (2.1 × 150 mm, 5 μm) coupled to a Dionex Ultimate 3000TM system and the column oven temperature was set to 25 °C for the gradient elution. A flow rate of 100 μL/min was used with the 10 mM ammonium carbonate in water (A), pH 9.0, and acetonitrile (B). The gradient profile was 80–20% B (0–30 min), 20–80% B (30–31 min), 80–80% B (31–42 min). Injection volume was set to 2 μL for all analyses (42 min total run time per injection). MS analyses were carried out by coupling the LC system to a Thermo Q Exactive HFTM mass spectrometer operating in heated electrospray ionization mode (HESI). Method duration was 30 min with polarity switching data-dependent Top 5 method for both positive and negative modes. Spray voltage for both positive and negative modes was 3.5 kV and the capillary temperature was set to 320s°C with a sheath gas rate of 35, aux gas of 10, and max spray current of 100 μA. The full MS scan for both polarities utilized 120,000 resolution with an AGC target of 3e^6^ and a maximum IT of 100 ms, and the scan range was from 67–1000 *m/z*. Tandem MS spectra for both positive and negative modes used a resolution of 15,000, AGC target of 1 e^5^, maximum IT of 50 ms, isolation window of 0.4 *m/z*, isolation offset of 0.1 *m/z*, fixed first mass of 50 *m/z*, and three-way multiplexed normalized collision energies (nCE) of 10, 35, 80. The minimum AGC target was 1e4 with an intensity threshold of 2e5. All data were acquired in profile mode. The data was analyzed using the R Package and web resource https://www.metaboanalyst.ca MetaboAnalyst 4.0^80^.

For the dopamine levels analysis, midbrain organoid samples were investigated with a reverse phase LCMS assay and dopamine was quantified across the samples. The samples were normalized by weight. Signals for dopamine were extracted by observing the peak height at *m/z* 154.0863. A background thresholding of 3 times the background signal plus 10,000 counts was used to determine the detection of dopamine in each sample. Finally, instrument performance was assessed using the internal standards added to the samples during extraction. Instrument mass accuracy was within tolerance (0.2 ppm), LC column performance was stable (0.39 min RT range) and internal standard response variability was 25% across the samples.

### Glycolysis stress test

Organoids were submitted to the glycolysis stress test in the Agilent Seahorse XF using the glycolysis stress test. Organoids were plated in a laminin-coated Seahorse XFe96 Spheroid Microplate on day 35 and analyzed on day 40 or day 200 after plating. The assays were done in XF base medium supplemented with B27 and N2. The data was acquired using the Seahorse Wave Desktop Software 2.6.1.

### Statistical analysis

All data were expressed in mean ± SEM. The statistical analysis was performed using Prism 9 (GraphPad) unless stated differently in the appropriate method section and the figures were created using Adobe Illustrator v 28. After experimental design appropriate null hypothesis testing and mean comparison, *P* values of <0.05 were considered significant. No statistical method was used to determine the sample size.

### Reporting summary

Further information on research design is available in the Nature Portfolio Reporting Summary linked to this article.

## Supplementary information

Supplementary Information

Description of Additional Supplementary Files

Supplementary Data 1

Reporting Summary

## Source data

Source Data

## Data Availability

The MS proteomics data have been deposited to the MassIVE repository with the dataset identifier MSV000090202. The data can be accessed directly via the link 10.25345/C5BV7B05F. The metadata of the experimental cases is described in STable 3. The metabolomics is submitted at the NIH Common Fund’s National Metabolomics Data Repository (NMDR) website, the Metabolomics Workbench, https://www.metabolomicsworkbench.org where it has been assigned Project ID (PR001491). The data can be accessed directly via its Project DOI: (10.21228/M80M7R). Source data are provided as a Source Data file. Source data are provided with this paper.

## Code availability

The analysis code is available at https://github.com/blanchardlab/Parfitt2023
