# Supplementary material for: Disruption of lysosomal proteolysis in astrocytes facilitates midbrain organoid proteostasis failure in an early-onset Parkinson’s disease model
Source: Nat Commun. 2024 Jan 10;15:447. doi: 10.1038/s41467-024-44732-2 (PMC10781970; doi:10.1038/s41467-024-44732-2)
Supplement: Supplementary file 1 — Supplementary Information [file 41467_2024_44732_MOESM1_ESM.pdf]

Disruption of lysosomal proteolysis in astrocytes facilitates midbrain organoid proteostasis failure in an early-onset PD model

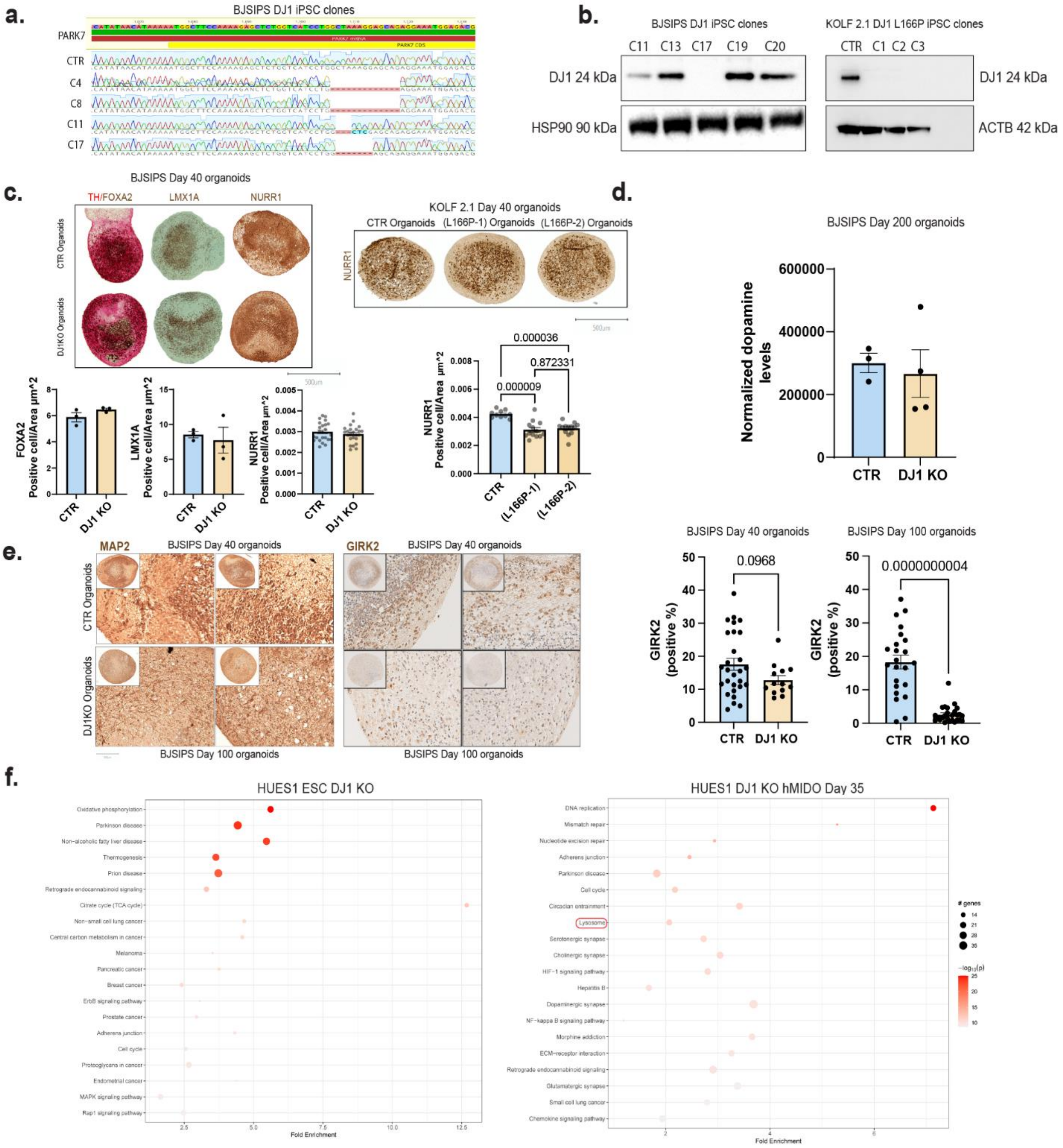

Figure S1. iPSC and midbrain organoid quality controls and validation. a. Representation of the Sanger sequencing chromatogram for DJ1 CRISPR clones in BJ-SIPS iPSC line. b. KO confirmation immunoblot for DJ1 CRISPR clones in BJ-SIPS and KOLF 2.1J iPSC lines. c.

Midbrain organoids staining and quantification of TH/FOXA2 (*n* = 3, Two-tailed t test was used for mean comparisons) and LMX1A (*n* = 3, Two-tailed t test was used for mean comparisons) markers at day 20 and NURR1 at day 40 positive cells for CTR and KO BJ-SIPS (*n* = 24, Two-tailed t test was used for mean comparisons) or KOLF 2.1J CTR (*n* = 10), DJ1 L166P KO1 (*n* = 13), and KO2 (*n* = 13, One-way ANOVA followed by Tukey's for the multiple comparisons test). d. Mass spectrometry dopamine quantification of midbrain organoids at day 200 (CTR, *n* = 3 and DJ1 KO, *n* = 4). e. Representative 40x images of hMIDO staining for MAP2 and GIRK2 at different time points and quantification of GIRK2 positive cells for day 40 (*n* = 3) and day 100 organoids (*n* = 3, Two-tailed t-test was used for mean comparisons). f. Pathway enrichment analysis in DJ1 KO generated from HUES1 iPSC and day 35 midbrain organoids. All data are represented in mean ± S.E.M, data points are individual organoids, and the p-value was reported on the graph highlighted comparison. All measurements were taken from distinct samples.

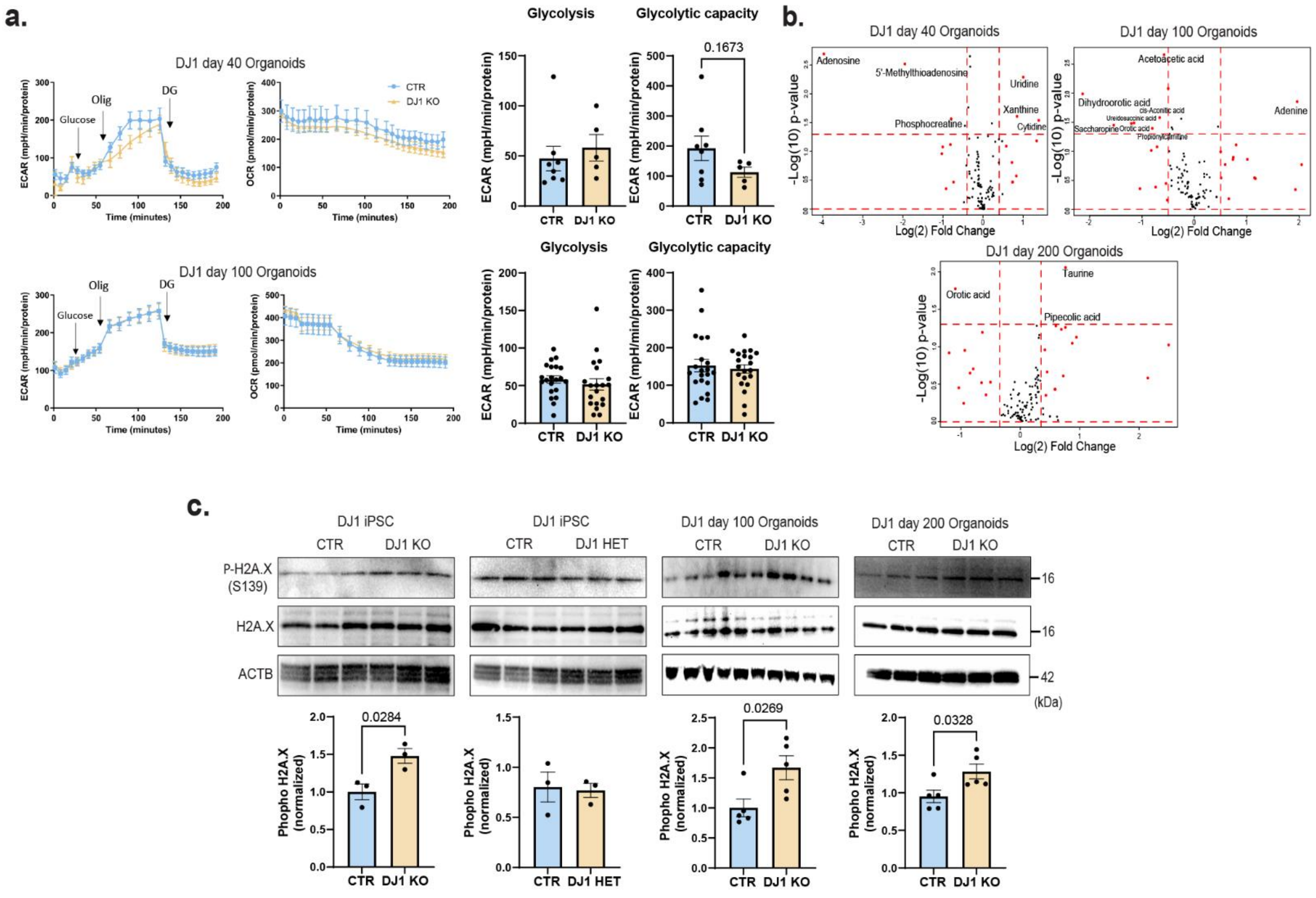

Figure S2. **Metabolic and DNA damage quantifications in midbrain organoids.** a. Glycolysis stress seahorse assay in day 40 (CTR, *n* = 8 and DJ1 KO, *n* = 5, Two-tailed t-test was used for mean comparisons) and day 100 (CTR, *n* = 21 and DJ1 KO, *n* = 20, Two-tailed t test was used for mean comparisons) midbrain organoids showing ECAR and OCR levels pre- glucose and after glucose, oligomycin, and DG treatments. b. Volcano plot representation of metabolomics panel for energy-related metabolites in day 40 (*n* = 6), 100 (*n* = CTR, *n* = 3 and DJ1 KO, *n* = 5), and 200 (CTR, *n* = 5 and DJ1 KO, *n* = 8) midbrain organoids. c. Immunoblots for native H2AX, phospho-H2AX (S139), and actin (ACTB) loading control for CTR (*n* = 3), DJ1 HET (*n* = 3), DJ1 KO iPSC (*n* = 3) and day 100 (*n* = 5) and 200 (*n* = 5) and respective CTR (*n* = 5) midbrain organoids (Two-tailed t test was used for mean comparisons). All data are represented in mean ± S.E.M, data points from seahorse assay are individual organoids, and data points for Wb are individual well differentiations. p-value was reported on the graph highlighted comparison. All measurements were taken from distinct samples.

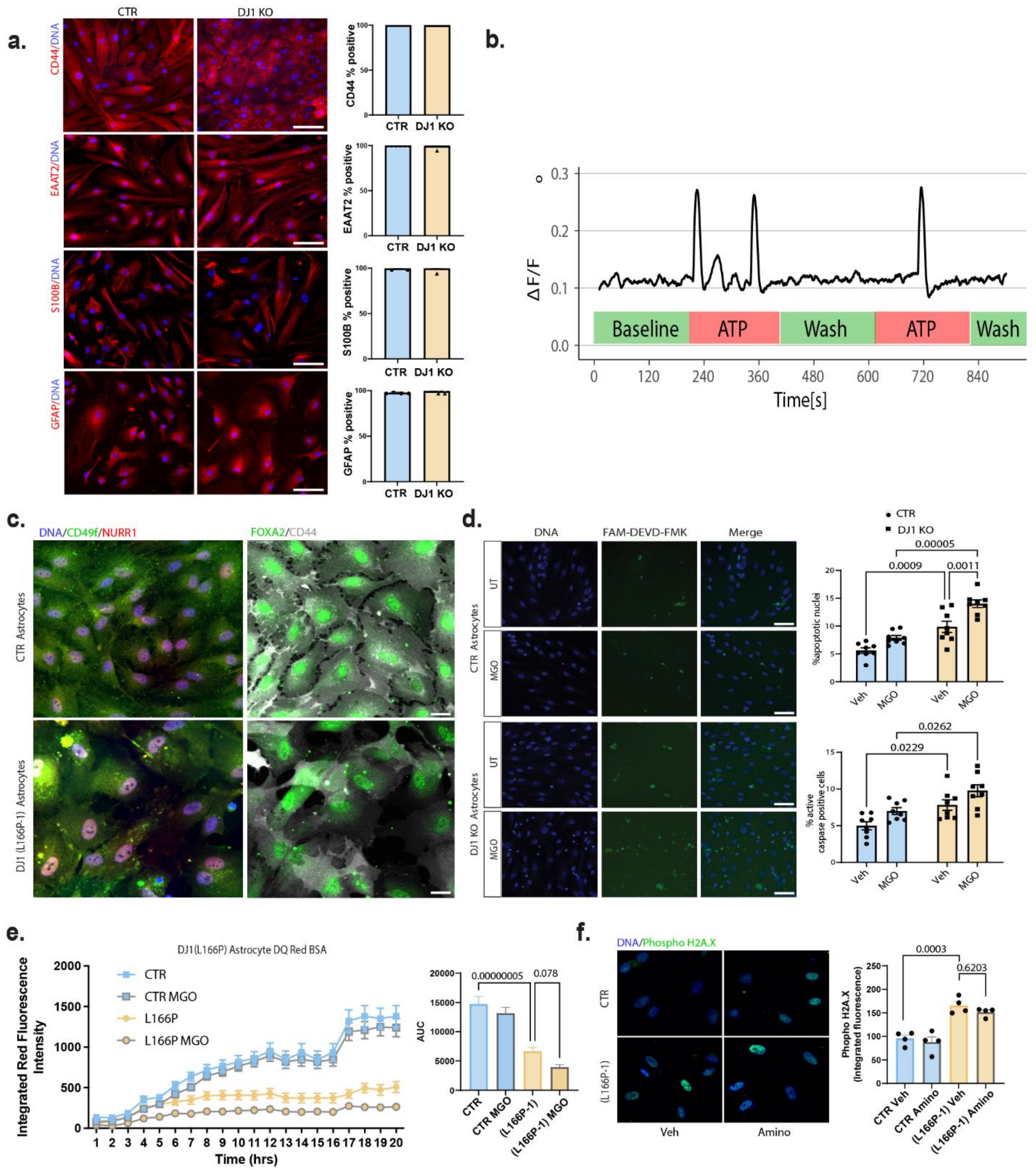

Figure S3. **Astrocyte markers and functional validation.** a. Immunostaining panel of CD44 (CTR, *n* = 8, DJ1 HET *n* = 12 and, DJ1 KO, *n* = 12), EAAT2 (CTR, *n* = 6, DJ1 HET *n* = 10 and, DJ1 KO, *n* = 10), S100B (CTR, *n* = 9, DJ1 HET *n* = 13 and, DJ1 KO, *n* = 13), and GFAP (CTR, *n* = 4, DJ1 HET *n* = 8 and, DJ1 KO, *n* = 8) counterstained with DAPI in midbrain astrocytes and % of positive cells (Two-tailed t test was used for mean comparisons). b. Astrocyte GCAMP8s calcium imaging δF/F trace before and after ATP stimulation. c. Immunostaining panel of midbrain

markers NUUR1 and FOXA2 co-stained with CD44. D. Poli-caspase reporter peptide FAM-DEVD-FMK staining in MGO treated or untreated CTR (*n* = 8) or DJ1 KO (*n* = 8) astrocytes counterstained with DAPI (Two-way ANOVA followed by Tukey's for the multiple comparisons test). d. Quantification of the standing showing percentage apoptotic nuclei and caspase-positive cells. e. DQBSA proteolysis live imaging assay of vehicle or MGO-treated astrocytes of CTR (*n* = 24) or DJ1 L166P (*n* = 24, Two-way ANOVA followed by Tukey's for the multiple comparisons test) genotypes. f. Immunostaining for phospho H2A.X in Amino-treated astrocytes (*n* = 4, Two-way ANOVA followed by Tukey's for the multiple comparisons test). Scale bars, 100 µm for A.; 20 µm for C; 50 µm for D. All data are represented in mean ± S.E.M, data points are individual organoids, and the p-value was reported on the graph highlighted comparison. All measurements were taken from distinct samples.

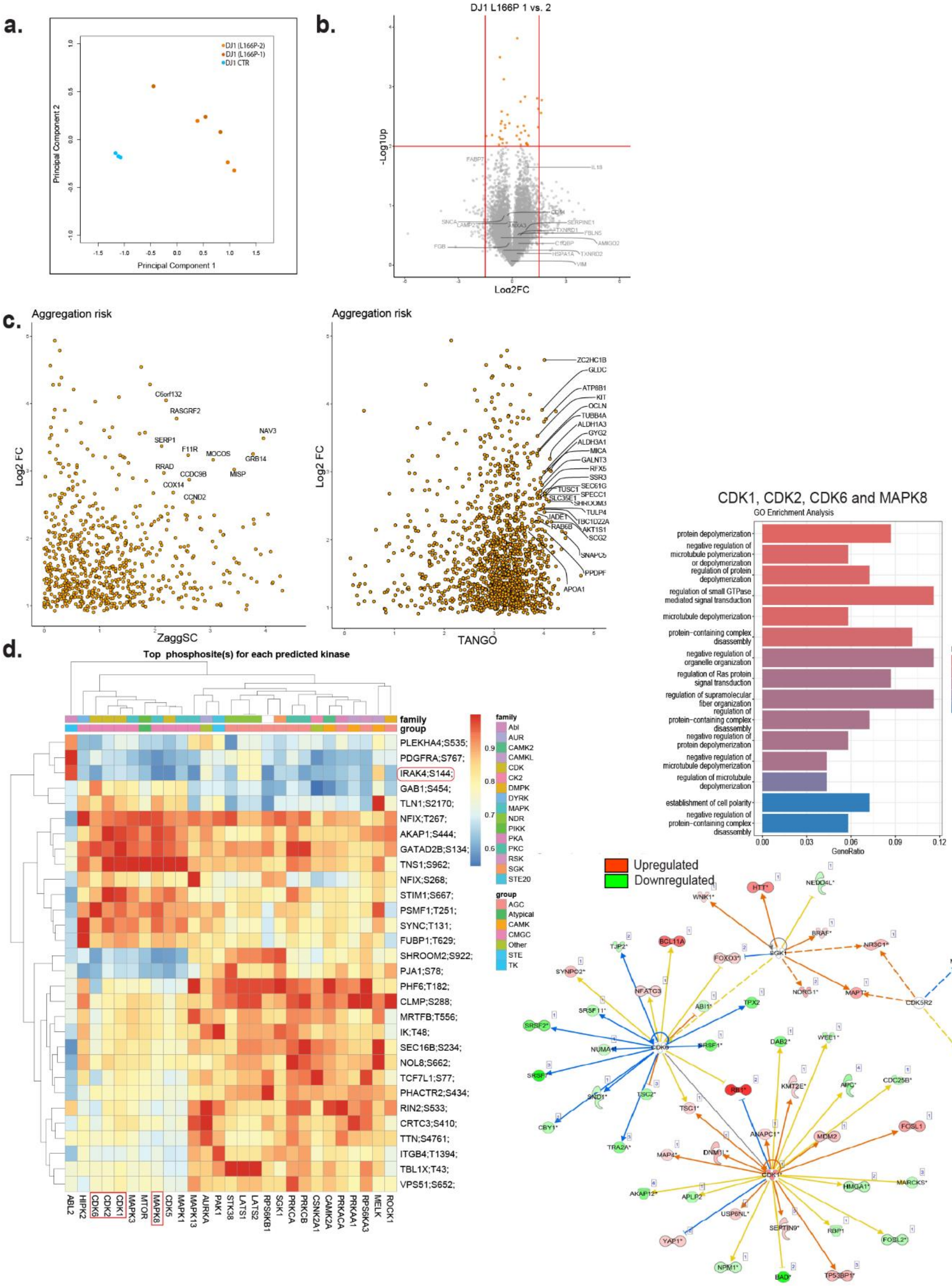
a.
DJ1 (L166P-2)
DJ1 (L166P-1)
DJ1 CTR
Principal Component 1
Principal Component 2
b.
DJ1 L166P 1 vs. 2
-Log10p
Log2FC
c.
Aggregation risk
Log2 FC
ZaggSC
TANGO
d.
Top phosphosite(s) for each predicted kinase
family
group
CDK1, CDK2, CDK6 and MAPK8
GO Enrichment Analysis
protein depolymerization
microtubule depolymerization
establishment of cell polarity
GeneRatio
p.adjust
Upregulated
Downregulated

Figure S4. Astrocyte proteomics quality control analysis and phosphoproteomics analysis a. PCA analysis of the TMT-labelled proteomics in KOLF 2.1J CTR (*n* = 3), L166P-1 (*n* = 3), and L166P-2 (*n* = 3) midbrain astrocytes (related to KN073-96 dataset). b. Volcano plot comparing the two DJ1 L166P clones highlighting selected proteins (related to KN073-96 dataset). c. Log2FC DJ1 L166P proteomics integration with aggregation risk scores for the human proteome ZaggSC and TANGO score showing selected proteins (related to KN073-96 dataset). d. Clustering plot of phospho-proteomics kinase activity prediction showing the top phosphosites, IPA Kinase/phosphosite network plots, and GO Enrichment analysis for CDK1, CDK2, CDK6, and MAPK8. All measurements were taken from distinct samples (related to KN043-066 and KN067-072 datasets).

a.

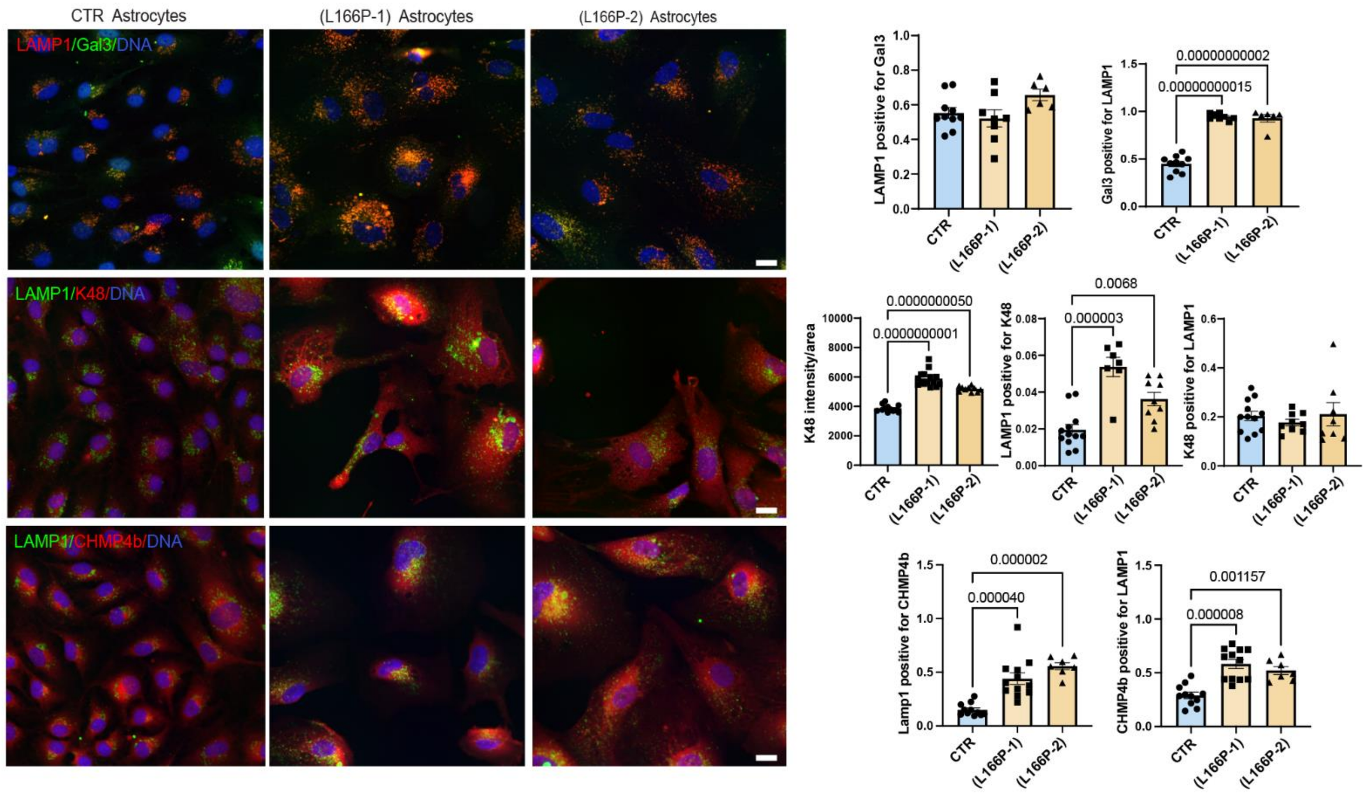

Figure S5. Astrocyte DJ1 loss of function lysosome damage and repair analysis. a. Immunostaining panel showing Gal3 co-stained with LAMP1 in KOLF 2.1J CTR, L166P-1, and L166P-2 midbrain astrocytes. Co-localization analysis of Gal3 co-stained with LAMP1(CTR, *n* = 10, L166P-1 *n* = 8 and, L166P-2, *n* = 6, One-way ANOVA followed by Tukey's for the multiple comparisons test). Immunostaining panel showing K48 Ub chain co-stained with LAMP1 in KOLF 2.1J CTR, L166P-1, and L166P-2 midbrain astrocytes. Co-localization analysis of K48 Ub chain

co-stained with LAMP1 (CTR, *n* = 12, L166P-1 *n* = 9 and, L166P-2, *n* = 8, One-way ANOVA followed by Tukey's for the multiple comparisons test). Immunostaining panel showing CHMP4b co-stained with LAMP1 in KOLF 2.1J CTR, L166P-1, and L166P-2 midbrain astrocytes. Co-localization analysis of CHMP4b co-stained with LAMP1 (CTR, *n* = 11, L166P-1 *n* = 12 and, L166P-2, *n* = 7, One-way ANOVA followed by Tukey's for the multiple comparisons test). Scale bars, 15 µm. All data are represented in mean ± S.E.M, data points are individual wells, and the p-value was reported on the graph highlighted comparison. All measurements were taken from distinct samples.

Case	Age	Sex	Clinical Diagnosis	Neuropathological Diagnosis
1	84	M	Parkinson's disease	Parkinson's disease
2	69	M	Parkinson's disease	Parkinson's disease
3	82	M	Parkinson's disease	Parkinson's disease
4	88	M	Parkinson's disease	Parkinson's disease
5	64	M	Parkinson's disease	Parkinson's disease
6	77	M	Parkinson's disease	Parkinson's disease
7	89	M	Pancreatic cancer	Control
8	65	F	ST-segment elevation myocardial infarction	Control
9	82	M	Cholecystitis	Control
10	77	F	Diabetes	Control
11	65	M	Acute myeloid leukemia	Control
Case	Age	Sex	Clinical Diagnosis	Neuropathological Diagnosis

Supplementary Table1. Clinical information on the PD patients cohort**.**

REAGENT or RESOURCE	SOURCE	IDENTIFIER
Antibodies/ Concentration		
Donkey anti-Mouse IgG, Alexa Fluor 488 (1:300)	Invitrogen	Cat#A32766
Donkey anti-Mouse IgG, Alexa Fluor 647 (1:300)	Invitrogen	Cat#A-31571
Donkey anti-Mouse IgG, Alexa Fluor 555 (1:300)	Invitrogen	Cat#A-31570
Donkey anti-Rabbit IgG, Alexa Fluor 488 (1:300)	Invitrogen	Cat#A-21206
Donkey anti-Rabbit IgG, Alexa Fluor 647 (1:300)	Invitrogen	Cat#A-31573
Peroxidase AffiniPure Goat Anti-Mouse IgG (H+L) (1:5000)	Jackson Laboratory	Cat#115-035-166; RRID: AB_2338511
Peroxidase AffiniPure Goat Anti-Mouse IgG (H+L) (1:5000)	Jackson Laboratory	Cat#111-035-144; RRID: AB_2307391
FOXA2 (1:100)	Abcam	Cat#Ab108422; RRID: AB_11157157
GAPDH (1:3000)	Abcam	Cat#Ab9485; RRID: AB_307275
GFAP (1:100 IF, 1:1000 WB)	Millipore	Cat#MAB360; RRID: AB_11212597
GFAP (1:10 IHC)	Roche	Cat#MAB360; RRID: N/A
TH (1:100 IF, 1:2000 WB)	Millipore	Cat#MAB318; RRID: AB_2201528
TH (1:100 IF, 1:2000 WB)	ABCAM	Cat#ab112;RRID: N/A

FOXA2 (1:500)	ABCAM	Cat#ab40874;RRID: N/A
PARK7/DJ1 (1:2000)	ABCAM	Cat#ab169520;RRID: N/A
LMX1A (1:500)	SIGMA	Cat#HPA030088;RRID: N/A
FOXA2 (1:500)	ABCAM	Cat#ab60721;RRID: N/A
alpha Synuclein (1:1000)	ABCAM	Cat#ab138501;RRID:AB_2537217
alpha Synuclein (Phosphorylated S129) (1:1000)	ABCAM	Cat#ab168381;RRID:AB_2728613
alpha Synuclein (Filament) (1:1000)	ABCAM	Cat#ab209538;RRID:AB_2714215
EAAT2 (1:100)	ABCAM	Cat#ab41621;RRID: N/A
LC3A/B (1:1000)	Cell Signaling	Cat#4108S;RRID: N/A
RAGE (1:1000)	ABCAM	Cat#ab37647;RRID: AB_777613
Histone H2A.X (1:1000)	Millipore	Cat#07-627; RRID:AB_2233033
CD49f (1:50)	Biolegend	Cat#313602;RRID:AB_345296
Methylglyoxal (MGO) (1:1000)	Cell Biolabs	Cat#STA-011,RRID: N/A
S100 – Beta (1:1000)	ABCAM	Cat#ab52642;RRID: AB_882426
LAMP1 (1:1000 WB, 1:50 IF)	ABCAM	Cat#ab25630;RRID: AB_470708
NR4A2 (NURR1) (1:100 IF, 1:3000 IHC)	SIGMA	Cat#N6413;RRID:AB_1841046

CHMP4B (1:100)	ptglab	Cat#13683-1-AP;RRID: N/A
Galectin-3 (Mac-2) (1:100)	Biolegend	Cat#125401;RRID:AB_1134237
CD44 (1:100)	ABCAM	Cat#ab157107;RRID:AB_2847859
Ubiquitin (Lys48-Specific), clone Apu2 (1:100 IF, 1:1000 Wb)	Millipore	Cat#05-1307;RRID:AB_1587578
Phospho-Histone H2A.X (Ser139), clone JBW301 (1:1000)	Millipore	Cat#05-636;RRID:AB_309864
oxDJ-1 (Cys106), clone M149 (1:400)	Millipore	Cat#MABN1773;RRID: N/A
β-Actin (C4) HRP (1:1000)	Santa Cruz	Cat#sc-47778;RRID:AB_2714189
β-Actin (AC-15) 1:5000)	Invitrogen	Cat#AM4302; RRID:AB_2536382
P62 (1:1000)	Progen	Cat#GP62-C;RRID:AB_2687531
GBA (1:1000)	Abnova	Cat#H00002629-M01;RRID:AB_464151
EAAT2 (1:100)	ABCAM	Cat#ab41621;RRID:AB_941782
Chemicals, peptides, and recombinant proteins		
Accutase® Cell Detachment Solution	Innovative Cell Technologies	Cat#AT104; RRID: AB_2869384
B-27™ Supplement (50×), Minus Vitamin A	Gibco	Cat#12587010; RRID: N/A
Bovine Albumin Fraction V (7.5% Solution)	Gibco	Cat#15260037; RRID: N/A

CHIR99021	Tocris	Cat#4423; RRID: N/A
DAPT	Cayman Chemical	Cat#13197; RRID: N/A
DB-cAMP/Dibutyryl-cAMP	Biolog	Cat#D 009; RRID: N/A
DirectPCR Lysis Reagent (Cell)	Viagen Biotech	Cat#302-C; RRID: N/A
DMEM/F-12, GlutaMAX™ Supplement	Gibco	Cat#10565018; RRID: N/A
Astrocyte Medium	Sciencell	Cat#1801; RRID: N/A
DMEM, High Glucose	Gibco	Cat#11965092; RRID: N/A
Geltrex™ LDEV-Free, hESC-Qualified, Reduced Growth Factor Basement Membrane Matrix	Gibco	Cat#A1413302; RRID: N/A
Hoechst 33342, Trihydrochloride, Trihydrate - 10 mg/mL Solution in Water	Invitrogen	Cat#H3570; RRID: N/A
Laminin Mouse Protein, Natural	Gibco	Cat#23017015; RRID: N/A
L-Ascorbic Acid (White Crystalline Powder)	Fisher Scientific	Cat#BP351-500; RRID: N/A
LDN193189	Stemgent	Cat#04-0074; RRID: N/A
Leibovitz's L-15 Medium	Gibco	Cat#11415064; RRID: N/A
N-2 Supplement (100×)	Gibco	Cat#17502048; RRID: N/A
Opti-MEM™ I Reduced Serum Medium	Gibco	Cat#31985062; RRID: N/A

PBS, pH 7.4 (-CaCl2, -MgCl2)	Gibco	Cat#10010023; RRID: N/A
Penicillin-Streptomycin (10,000 U/mL)	Gibco	Cat#15140122; RRID: N/A
Poly-L-Ornithine Hydrobromide	Sigma-Aldrich	Cat#P3655; RRID: N/A
Proteinase K Solution (20 mg/mL)	Viagen Biotech	Cat#501-PK; RRID: N/A
Purmorphamine	STEMCELL Technologies	Cat#72204; RRID: N/A
Puromycin Dihydrochloride	Gibco	Cat#A1113803; RRID: N/A
Recombinant Human BDNF Protein	R&D Systems	Cat#248-BD; RRID: N/A
Recombinant Human CNTF Protein	Peprotech	Cat#450-13;RRID: N/A
Recombinant Human GDNF Protein	R&D Systems	Cat#212-GD; RRID: N/A
SAG	Cayman Chemical	Cat#11914; RRID: N/A
SB431542	Stemgent	Cat#04-0010-10; RRID: N/A
StemFlex™ Medium	Gibco	Cat#A3349401; RRID: N/A
Thiazovivin	Selleck Chemicals	Cat#S1459; RRID: N/A
Trypan Blue Solution, 0.4%	Gibco	Cat#15250061; RRID: N/A
Aminoguanidine hydrochloride	Sigma	Cat#1937-19-5; RRID: N/A
Bafilomycin A1 from Streptomyces griseus	Sigma	Cat#B1793; RRID: N/A

(R)-MG-132	Selleck Chemicals	Cat#S2619; RRID: N/A
Non-essential Amino Acids (NEAA)	Gibco	Cat#11140050;RRID: N/A
GlutaMax Supplement	Gibco	Cat#35050061;RRID: N/A
Triton X-100	Sigma	Cat# T9284;RRID: N/A
RIPA buffer	Sigma	Cat# R0278;RRID: N/A
DQ™ Red BSA	ThermoFisher	Cat#D12051;RRID: N/A
Y-27632 Dihydrochloride	Tocris	Cat#1254; RRID: N/A
Critical commercial assays		
Seahorse XF Glycolysis Stress Test Kit	Agilent	Cat#103020-100;RRID: N/A
Image-iT™ LIVE Red and Green Caspase Apoptosis Detection Kits for microscopy	ThermoFisher	Cat#I35101;RRID: N/A
Lipofectamine™ Stem Transfection Reagent	Invitrogen	Cat#STEM00003; RRID: N/A
PROTEOSTAT	Enzo	Cat#ENZ-51023;RRID: N/A
P3 Primary Cell 4D-Nucleofector™ X Kit S	Lonza	Cat#V4XP-3032; RRID: N/A
Experimental models: Cell lines		
Human: BJ SiPS-D induced pluripotent stem cells	Harvard University	Cat#BJ SiPS-D: RRID: CVCL_X741
KOLF2.1J	Jackson Laboratory	RRID: N/A

KOLF2.1J DJ1 L166P 1	Jackson Laboratory	RRID: N/A
KOLF2.1J DJ1 L166P 2	Jackson Laboratory	RRID: N/A
Human: BJ SiPS-D induced pluripotent stem cells Dj1 HET #11	This paper	RRID: N/A
Human: BJ SiPS-D induced pluripotent stem cells Dj1 KO #17	This paper	RRID: N/A
Oligonucleotides		
PARK7 _L166P F ATGCATACCCGCCTC CATTACGTTG	This paper	RRID: N/A
PARK7 _L166P R ATAAGCAGAGAAAAT CACAAGCCTC	This paper	RRID: N/A
PARK7 _L166P seq AATGGATTCCTAA CGGCCTG	This paper	RRID: N/A
PARK7 R ATGGCTAAAAATCGATGTG G	This paper	RRID: N/A
PARK7 F TGGGGTATCTCAGGGTTG CA	This paper	RRID: N/A
Recombinant DNA		
Plasmid: pTAHR TH-p2a-TD:Tomato (floxed selection Puro)	Ahfeldt et al., 2020; Addgene	Cat#135814; RRID: N/A
CRISPR 1: pTACR TH1-p2aGFP	Ahfeldt et al., 2020; Addgene	Cat#135815; RRID: N/A

CRISPR 2: pTACR TH2-p2aGFP	Ahfeldt et al., 2020; Addgene	Cat#135816; RRID: N/A
Plasmid: pCAG-Cre:GFP	Matsuda and Cepko, 2007; Addgene	Cat#13776; RRID: Addgene_13776
Software and algorithms		
CellProfiler	https://cellprofiler.org/	RRID:SCR_007358
Rstudio	https://www.rstudio.com/	RRID:SCR_000432
Ingenuity Pathway Analysis (IPA)	Qiagen	RRID:SCR_008653
QuPath	https://QuPath.github.io/	RRID:SCR_018257
Pathfinder	https://github.com/egeulgen/pathfindR	Ulgen et al., 2019
PhosR R packages	https://github.com/PYangLab/PhosR	Kim et al., 2021
MetaboAnalyst 4.0	https://www.metaboanalyst.ca	RRID:SCR_015539
Fiji	https://fiji.sc/	Chong et al., 2019; RRID:SCR_002285
Other		
4D-Nucleofector™ Core Unit	Lonza	Cat#AAF-1002B; RRID: N/A
Cell Culture Microplate, 96-well, PS, F-bottom (Chimney Well), µClear®, Black, CELLCOAT®, Poly-L-Lysine, Lid with Condensation Rings, 5 pcs./bag	Greiner	Cat#655936; RRID: N/A
Corning® 125 mL Disposable Spinner Flask	Corning	Cat#3152; RRID: N/A

with 70 mm Top Cap and 2 Angled Sidearms, Sterile		
Corning® Costar® Ultra-Low Attachment Multiple Well Plate	MilliporeSigma	Cat#CLS3471; RRID: N/A
Countess™ Cell Counting Chamber Slides	Invitrogen	Cat#C10228; RRID: SCR_019815
Countess™ II FL Automated Cell Counter	Invitrogen	Cat#AMQAF1000; RRID: N/A
Falcon® Round-Bottom Tubes with Cell Strainer Cap, 5 mL	STEMCELL Technologies	Cat#100-0087; RRID: N/A
Fisherbrand™ Disposable Borosilicate Glass Pasteur Pipets	Fisher Scientific	Cat#13-678-20D; RRID: N/A
Fisherbrand™ Sterile Cell Strainers (70 µm)	Fisher Scientific	Cat#22-363-548; RRID: N/A
pluriStrainer® 300 µm, 25 pcs. – Sterile (Cell Strainer)	pluriSelect	Cat#43-50300-03; RRID: N/A
pluriStrainer® 500 µm, 25 pcs. – Sterile (Cell Strainer)	pluriSelect	Cat#43-50500-03; RRID: N/A
Stirrers, Magnetic, Nine-position, Dura-Mag	Chemglass Life Sciences	Cat#CLS-4100-09; RRID: N/A
Variable Speed 2D Rocker	USA Scientific	Cat#2527-2000; RRID: N/A

Supplementary Table2. List of reagents.

Exp1_AO

4plex TMTpro proteomics dataset:

Sample	Channel	Clone	Cell type	Lysate preparation
WT_1	132c	KOLF 2.1J background	Midbrain patterned astrocytes of 100 days	RIPA
WT_2	133n	KOLF 2.1J background	Midbrain patterned astrocytes of 100 days	RIPA
L166P_1	133c	L166P-1/KOLF 2.1J background	Midbrain patterned astrocytes of 100 days	RIPA
L166P_2	134n	L166P-1/KOLF 2.1J background	Midbrain patterned astrocytes of 100 days	RIPA

Source files	Experiment
KN043-066	Total proteome (FAIMS-MS3)
KN067-072	Phospho proteome (FAIMS-MS2)

Exp2_AO

9plex TMT (FAIMS-hrMS2) proteomics dataset:

Sample	Channel	Clone	Cell type	Lysate preparation
WT_1	126	KOLF 2.1J background	Midbrain patterned astrocytes of 100 days	RIPA
WT_2	127n	KOLF 2.1J background	Midbrain patterned astrocytes of 100 days	RIPA
WT_3	127c	KOLF 2.1J background	Midbrain patterned astrocytes of 100 days	RIPA
L166P_A_1	128n	L166P-1/KOLF 2.1J background	Midbrain patterned astrocytes of 100 days	RIPA
L166P_A_2	128c	L166P-1/KOLF 2.1J background	Midbrain patterned astrocytes of 100 days	RIPA
L166P_A_3	129n	L166P-1/KOLF 2.1J background	Midbrain patterned astrocytes of 100 days	RIPA
L166P_B_1	129c	L166P-2/KOLF 2.1J background	Midbrain patterned astrocytes of 100 days	RIPA
L166P_B_2	130n	L166P-2/KOLF 2.1J background	Midbrain patterned astrocytes of 100 days	RIPA
L166P_B_3	130c	L166P-2/KOLF 2.1J background	Midbrain patterned astrocytes of 100 days	RIPA

Source files	Experiment
KN073-96	Total proteome(FAIMS-MS2)

Supplementary Table3. Metadata for the proteomics and phosphoproteomics experiments.
