# Supplementary material for: Disruption of lysosomal proteolysis in astrocytes facilitates midbrain organoid proteostasis failure in an early-onset Parkinson’s disease model
Source: Nat Commun. 2024 Jan 10;15:447. doi: 10.1038/s41467-024-44732-2 (PMC10781970; doi:10.1038/s41467-024-44732-2)
Supplement: Supplementary file 2 — Description of Additional Supplementary Files [file 41467_2024_44732_MOESM2_ESM.pdf]

Description of Additional Supplementary Files

File Name: Supplementary Data 1
Description: Pathway analysis of the proteomics datasets.
