# Supplementary material for: Disruption of lysosomal proteolysis in astrocytes facilitates midbrain organoid proteostasis failure in an early-onset Parkinson’s disease model
Source: Nat Commun. 2024 Jan 10;15:447. doi: 10.1038/s41467-024-44732-2 (PMC10781970; doi:10.1038/s41467-024-44732-2)
Supplement: Supplementary file 4 — Reporting Summary [file 41467_2024_44732_MOESM4_ESM.pdf]

nature portfolio

Corresponding author(s): Tim Ahfeldt, Joel Blanchard and Gustavo Parfitt

Last updated by author(s): 2023/12/06

Reporting Summary

Nature Portfolio wishes to improve the reproducibility of the work that we publish. This form provides structure for consistency and transparency in reporting. For further information on Nature Portfolio policies, see our Editorial Policies and the Editorial Policy Checklist.

Statistics

For all statistical analyses, confirm that the following items are present in the figure legend, table legend, main text, or Methods section.

n/a	Confirmed	
☐	☒	The exact sample size (*n*) for each experimental group/condition, given as a discrete number and unit of measurement
☐	☒	A statement on whether measurements were taken from distinct samples or whether the same sample was measured repeatedly
☐	☒	The statistical test(s) used AND whether they are one- or two-sided<br>*Only common tests should be described solely by name; describe more complex techniques in the Methods section.*
☒	☐	A description of all covariates tested
☐	☒	A description of any assumptions or corrections, such as tests of normality and adjustment for multiple comparisons
☐	☒	A full description of the statistical parameters including central tendency (e.g. means) or other basic estimates (e.g. regression coefficient) AND variation (e.g. standard deviation) or associated estimates of uncertainty (e.g. confidence intervals)
☐	☒	For null hypothesis testing, the test statistic (e.g. *F*, *t*, *r*) with confidence intervals, effect sizes, degrees of freedom and *P* value noted<br>*Give P values as exact values whenever suitable.*
☒	☐	For Bayesian analysis, information on the choice of priors and Markov chain Monte Carlo settings
☒	☐	For hierarchical and complex designs, identification of the appropriate level for tests and full reporting of outcomes
☒	☐	Estimates of effect sizes (e.g. Cohen's *d*, Pearson's *r*), indicating how they were calculated

Our web collection on statistics for biologists contains articles on many of the points above.

Software and code

Policy information about availability of computer code

Data collection	Seahorse Wave Desktop Software 2.6.1, QExactive HF, Comet software 2019.01 rev. 5, HCS Studio 4.0, Nikon Elements software AR 5.20.01, Incucyte® software v2020C, Aperio software 8.
Data analysis	Microsoft Excel v16, R 4.0.2, Monocle v0.4, Perseus, QIAGEN Ingenuity Pathway Analysis (IPA) software v1.0, pathfindR 1.6.1 , PhosR R v1.12 , MetaboAnalyst 4.0 R packages, CellProfiller v3, ImageJ v1.53 , Prism 9, QuPath (version 0.2.3), Incucyte® software v2020C.<br>Data analysis code for R is available at https://github.com/blanchardlab/Parfitt2023

For manuscripts utilizing custom algorithms or software that are central to the research but not yet described in published literature, software must be made available to editors and reviewers. We strongly encourage code deposition in a community repository (e.g. GitHub). See the Nature Portfolio guidelines for submitting code & software for further information.

Data

Policy information about availability of data

All manuscripts must include a data availability statement. This statement should provide the following information, where applicable:

- Accession codes, unique identifiers, or web links for publicly available datasets
- A description of any restrictions on data availability
- For clinical datasets or third party data, please ensure that the statement adheres to our policy

The data from this study is available in the Source data file.

Data and code availability
The MS proteomics data have been deposited to the MassIVE repository with the dataset identifier MSV000090202. The metadata of the experimental cases is described in Table 3.

The MS proteomics data have been deposited to the MassIVE repository with the dataset identifier MSV000090202. The metadata of the experimental cases is described in Table 3.

The metabolomics is submitted at the NIH Common Fund's National Metabolomics Data Repository (NMDR) website, the Metabolomics Workbench, https://www.metabolomicsworkbench.org where it has been assigned Project ID (PR001491). The data can be accessed directly via its Project DOI: (10.21228/M80M7R).

Research involving human participants, their data, or biological material

Policy information about studies with human participants or human data. See also policy information about sex, gender (identity/presentation), and sexual orientation and race, ethnicity and racism.

Reporting on sex and gender	We report the sex of the subjects which biological material was collected. We included the most sex diversity brain tissue available.
Reporting on race, ethnicity, or other socially relevant groupings	Due to the small cohort of subjects available, we do not include information on race, ethnicity, or other socially relevant groupings.
Population characteristics	The Parkinson's disease and Control subjects were aged-matched, with the age interval from 65 to 89 in the Controls and 64 to 88 in the Parkinson's disease. The clinical diagnoses of the control subjects had no biases, with all having different diagnoses.
Recruitment	Research with de-identified autopsy material does not meet the federal regulatory definition of human subject research as defined in 45 CFR part 46 and is otherwise exempt.
Ethics oversight	Research with de-identified autopsy material does not meet the federal regulatory definition of human subject research as defined in 45 CFR part 46 and is otherwise exempt.

Note that full information on the approval of the study protocol must also be provided in the manuscript.

Field-specific reporting

Please select the one below that is the best fit for your research. If you are not sure, read the appropriate sections before making your selection.

[x] Life sciences [ ] Behavioural & social sciences [ ] Ecological, evolutionary & environmental sciences

For a reference copy of the document with all sections, see nature.com/documents/nr-reporting-summary-flat.pdf

Life sciences study design

All studies must disclose on these points even when the disclosure is negative.

Sample size	No statistical was used to predetermined the sample size. We used other studies from the field to guide the choice of the sample size (https://doi.org/10.1038/s41586-022-05439-;https://doi.org/10.1016/j.stemcr.2019.12.005).
Data exclusions	Some data points were excluded based on outlier statistical analysis and artifacts present during the data acquisition. In addition, differentiations that did not pass quality control of a panel of antibodies staining were not included.
Replication	The main results of the papers were replicated in at lest 3 differentiations. All the data points represent different organoids, wells of a differentiation or human subjects.
Randomization	Samples were assigned based on genotype. In the case of treatments applied to the samples, the wells were assigned to prevent the position bias on the plate.
Blinding	The investigators were not blinded to group allocations for imaging collections. However, the data was collected in automated microscopes for fluorescence and IHC experiments. With the exception of imaging where the data analysis was done blind to the groups identification. The investigators were not blinded to group allocations for other experiments. The analysis pipelines were standard and patronized prior to the data acquisition.

Reporting for specific materials, systems and methods

We require information from authors about some types of materials, experimental systems and methods used in many studies. Here, indicate whether each material, system or method listed is relevant to your study. If you are not sure if a list item applies to your research, read the appropriate section before selecting a response.

Materials & experimental systems

n/a	Involved in the study
☐	☒ Antibodies
☐	☒ Eukaryotic cell lines
☒	☐ Palaeontology and archaeology
☒	☐ Animals and other organisms
☐	☒ Clinical data
☒	☐ Dual use research of concern
☒	☐ Plants

Methods

n/a	Involved in the study
☒	☐ ChIP-seq
☒	☐ Flow cytometry
☒	☐ MRI-based neuroimaging

Antibodies

Antibodies used

Antibody SOURCE IDENTIFIER
Donkey anti-Mouse IgG, Alexa Fluor 488 Invitrogen Cat#A32766
Donkey anti-Mouse IgG, Alexa Fluor 647 Invitrogen Cat#A-31571
Donkey anti-Mouse IgG, Alexa Fluor 555 Invitrogen Cat#A-31570
Donkey anti-Rabbit IgG, Alexa Fluor 488 Invitrogen Cat#A-21206
Donkey anti-Rabbit IgG, Alexa Fluor 647 Invitrogen Cat#A-31573
Peroxidase AffiniPure Goat Anti-Mouse IgG Jackson Laboratory Cat#115-035-166;RRID: AB_2338511
Peroxidase AffiniPure Goat Anti-Mouse IgG Jackson Laboratory Cat#111-035-144; RRID: AB_2307391
GFAP Roche Cat#MAB360; RRID: N/A
FOXA2 Abcam Cat#Ab108422; RRID: AB_11157157
GAPDH Abcam Cat#Ab9485; RRID: AB_307275
GFAP Millipore Cat#MAB360; RRID: AB_11212597
TH Millipore Cat#MAB318; RRID: AB_2201528
TH ABCAM Cat#ab112;RRID: N/A
FOXA2 ABCAM Cat#ab40874;RRID: N/A
PARK7/DJ1 ABCAM Cat#ab169520;RRID: N/A
LMX1A SIGMA Cat#HPA030088;RRID: N/A
FOXA2 ABCAM Cat#ab60721;RRID: N/A
TH Millipore Cat#MAB318; RRID:AB_2201528
alpha Synuclein ABCAM Cat#ab138501;RRID:AB_2537217
alpha Synuclein (Phosphorylated S129) ABCAM Cat#ab168381;RRID:AB_2728613
alpha Synuclein (Filament) ABCAM Cat#ab209538;RRID:AB_2714215
EAAT2 ABCAM Cat#ab41621;RRID: N/A
LC3A/B Cell Signaling Cat#4108S;RRID: N/A
RAGE ABCAM Cat#ab37647;RRID:AB_777613
Histone H2A.X Millipore Cat#07-627; RRID:AB_2233033
CD49f Biolegend Cat#313602;RRID:AB_345296
Methylglyoxal (MGO) Cell Biolabs Cat#STA-011,RRID: N/A
S100 - Beta ABCAM Cat#ab52642;RRID:AB_882426
LAMP1 ABCAM Cat#ab25630;RRID:AB_470708
LC3B Cell Signaling 3868S;RRID: N/A
NR4A2 (NURR1) SIGMA Cat#N6413;RRID:AB_1841046
CHMP4B ptglab Cat#13683-1-AP;RRID: N/A
Galectin-3 (Mac-2) Biolegend Cat#125401;RRID:AB_1134237
CD44 ABCAM Cat#ab157107;RRID:AB_2847859
Ubiquitin (Lys48-Specific), clone Apu2 Millipore Cat#05-1307;RRID:AB_1587578
Phospho-Histone H2A.X (Ser139), clone JBW301 Millipore Cat#05-636;RRID:AB_309864
oxDJ-1 (Cys106), clone M149 Millipore Cat#MABN1773;RRID: N/A
β-Actin (C4) HRP Santa Cruz Cat#sc-47778;RRID:AB_2714189
β-Actin (AC-15) Invitrogen Cat#AM4302; RRID:AB_2536382
P62 Progen Cat#GP62-C;RRID:AB_2687531
GBA Abnova Cat#H00002629-M01;RRID:AB_464151
EAAT2 ABCAM Cat#ab41621;RRID:AB_941782

Validation

All the antibodies used in the study were commercially available and the more information is available in the manufacturer website.

Eukaryotic cell lines

Policy information about cell lines and Sex and Gender in Research

Cell line source(s)	BJSIPS TH-Tdtomato DJ1 WT<br>BJSIPS TH-Tdtomato DJ1 HET<br>BJSIPS TH-Tdtomato DJ1 KO<br>The iPSC lines used for the generation of the DJ1 KO were from the BJSIPS background and the guides as described in (Ahfeldt et al., 2020). The cell lines were deposited at Wicell.<br><br>KOLF2.1J<br>KOLF2.1J DJ1 L166P clone 1<br>KOLF2.1J DJ1 L166P clone 2<br><br>Source Bill Skarnes, iPSC Neurodegenerative Disease Initiative (iNDI), the Center for Alzheimer's and Related Dementias (CARD) and the ASAP consortium.
Authentication	The cells lines were authenticated using western blot and Sanger sequencing.
Mycoplasma contamination	The cells tested negative for micoplasma and were tested every two months.
Commonly misidentified lines<br>(See ICLAC register)	No commonly misidentified cell lines were used in this study.

Clinical data

Policy information about clinical studies

All manuscripts should comply with the ICMJEguidelines for publication of clinical research and a completedCONSORT checklist must be included with all submissions.

Clinical trial registration	N/A
Study protocol	N/A
Data collection	N/A
Outcomes	N/A
